# Biodiversity and host-parasite cophylogeny of *Sphaerospora* (*sensu stricto*) (Cnidaria: Myxozoa)

**DOI:** 10.1186/s13071-018-2863-z

**Published:** 2018-06-15

**Authors:** Sneha Patra, Pavla Bartošová-Sojková, Hana Pecková, Ivan Fiala, Edit Eszterbauer, Astrid S. Holzer

**Affiliations:** 1 Institute of Parasitology, Biology Centre, Czech Academy of Sciences, 37005 České Budějovice, Czech Republic; 2 Faculty of Science, University of South Bohemia, 37005 České Budějovice, Czech Republic; 3 Institute for Veterinary Medical Research, Centre for Agricultural Research, Budapest, H-1143 Hungary

**Keywords:** Myxozoa, *Sphaerospora sensu stricto*, Taxonomy, Phylogeny, Teleost, Host-parasite codiversification

## Abstract

**Background:**

Myxozoa are extremely diverse microscopic parasites belonging to the Cnidaria. Their life-cycles alternate between vertebrate and invertebrate hosts, predominantly in aquatic habitats. Members of the phylogenetically well-defined *Sphaerospora* (*sensu stricto*) clade predominantly infect the urinary system of marine and freshwater fishes and amphibians. Sphaerosporids are extraordinary due to their extremely long and unique insertions in the variable regions of their *18S* and *28S* rDNA genes and due to the formation of motile proliferative stages in the hosts' blood. To date, DNA sequences of only 19 species have been obtained and information on the patterns responsible for their phylogenetic clustering is limited.

**Methods:**

We screened 549 fish kidney samples from fish of various geographical locations, mainly in central Europe, to investigate sphaerosporid biodiversity microscopically and by *18S* rDNA sequences. We performed multiple phylogenetic analyses to explore phylogenetic relationships and evolutionary trends within the *Sphaerospora* (*s.s.*) clade, by matching host and habitat features to the resultant *18S* rDNA trees. The apparent co-clustering of species from related fish hosts inspired us to further investigate host-parasite co-diversification, using tree-based (CoRE-PA) and distance-based (ParaFit) methods.

**Results:**

Our study considerably increased the number of *18S* rDNA sequence data for *Sphaerospora* (*s.s.*) by sequencing 17 new taxa. Eight new species are described and one species (*Sphaerospora diminuta* Li & Desser, 1985) is redescribed, accompanied by sufficient morphological data. Phylogenetic analyses showed that sphaerosporids cluster according to their vertebrate host order and habitat, but not according to geography. Cophylogenetic analyses revealed a significant congruence between the phylogenetic trees of sphaerosporids and of their vertebrate hosts and identified Cypriniformes as a host group of multiple parasite lineages and with high parasite diversity.

**Conclusions:**

This study significantly contributed to our knowledge of the biodiversity and evolutionary history of the members of the *Sphaerospora* (*s.s.*) clade. The presence of two separate phylogenetic lineages likely indicates independent historical host entries, and the remarkable overlap of the larger clade with vertebrate phylogeny suggests important coevolutionary adaptations. Hyperdiversification of sphaerosporids in cypriniform hosts, which have undergone considerable radiations themselves, points to host-driven diversification.

**Electronic supplementary material:**

The online version of this article (10.1186/s13071-018-2863-z) contains supplementary material, which is available to authorized users.

## Background

The Myxozoa Grassé, 1970 is a diverse group of cnidarian fish parasites, which is composed of over 2400 species [1]. The genus *Sphaerospora* Thélohan, 1892 consists of 102 described species with *18S* rDNA sequences presently available for only 24 taxa (NCBI database, December 2017). Myxospores from vertebrate hosts are spherical to subspherical with two identical valves, a single binucleated or 2–12 uninucleated sporoplasms and two subspherical polar capsules situated perpendicular to the sutural plane [2, 3]. *Sphaerospora* (*sensu stricto*) (*s.s.*) is a phylogenetic clade of myxozoans that hosts the type-species *Sphaerospora elegans* Thélohan, 1892 and 18 other species considered “true sphaerosporids” [4–7]. Extremely long insertions in the variable regions of *18S* and *28S* rDNA genes are exclusive features of this unique clade of myxozoans [4, 5, 8] which define the clade. *Sphaerospora molnari* Lom, Dyková, Pavlasková & Grupcheva, 1983 possesses one of the longest eukaryotic *18S* rDNA sequences (3.7 kb) [9].

Most of the members of *Sphaerospora* (*s.s.*) are coelozoic in the excretory system (predominantly renal tubules), only two histozoic taxa, i.e. *Sphaerospora fugu* (Tun, Yokoyama, Ogawa & Wakayabashi, 2000) and *S. molnari*, have been sequenced to date. Members of *Sphaerospora* (*s.s.*) are believed to have similar life-cycle strategy like other myxozoans that alternate between vertebrate and invertebrate hosts [5, 10]. Life-cycles were described for *Sphaerospora truttae* Fischer-Scherl, El-Matbouli & Hoffmann, 1986 [11] and *Sphaerospora dykovae* Gunter & Adlard, 2010 [12] but that of *S. truttae* was later shown to be incorrect as the alternate spore stages from the two hosts did not have identical *18S* rDNA sequences [13], while the invertebrate life-cycle stage of *S. dykovae* still requires molecular confirmation [14]. Vertebrate hosts for sphaerosporids are marine and freshwater teleost fishes as well as amphibians [4, 5, 15].

The evolution of parasites and their hosts is shaped by their reciprocal influence and it was recently demonstrated that myxozoans and their invertebrate hosts show a high degree of phylogenetic congruence, likely because the latter represent the host group that was first acquired by myxozoans [16]. Cophylogenetic signal between myxozoans and their vertebrate hosts is more obscured as the coevolutionary history of reciprocal adaptation of myxozoans and their intermediate vertebrate hosts is much shorter and is received as a “mixed signal” of invertebrate and vertebrate cophylogeny [16]. Myxozoans are potentially the fastest evolving metazoans on the planet [16–20], with the most radical nucleotide variability found in true sphaerosporids. This group has also a wide range of vertebrate hosts, making them an especially interesting case for cophylogenetic studies. Lack of data for sphaerosporids in a recent study evaluating reciprocal dependencies of the phylogenies of myxozoans and their vertebrate hosts led to inconsistent results in this clade [16].

In the present study, we screened fish kidneys specifically for sphaerosporids, provide descriptions of a wide spectrum of *Sphaerospora* spp. accompanied by data on host specificity and *18S* rDNA sequences that significantly enrich the dataset for phylogenetic and cophylogenetic analyses. We reinvestigated host-parasite codivergence using an extended sphaerosporid dataset, allowing for a detailed analysis of interdependent phylogenies and recreation of the evolutionary history of this special group of myxozoan parasites.

## Methods

### Sample collection and parasite morphology

In total, 549 fishes belong to 28 species (542 from freshwater and 7 from marine habitats) were collected from various localities, mostly in the Czech Republic (Additional file 1: Table S1), between 2012 and 2017. The highest number of fishes belongs to the order Cypriniformes (424 fish, 16 species) and Perciformes (82 fish, 3 species). All captured fish were euthanized by an overdose of clove oil followed by neural pithing. Kidney samples from all fishes were collected using sterile scissors and scalpel blades. Kidney samples were freshly squashed on grease-free glass slides and checked under an Olympus BX51 microscope, followed by digital documentation of kidneys containing presporogonic stages and *Sphaerospora* spores, using an Olympus DP70 camera. Preliminary species identification was performed referring to published guidelines [3, 21, 22]. Spores were measured on digital images using ImageJ 1·48q (Wayne Rasband, http://imagej.nih.gov/ij, Java 1·7·0_45; 64 bit) in reference to graticule measurements. Spore measurements (in μm) include spore length (L) and thickness (T), polar capsule length (PL) and width (PW), as well as plasmodium length and width given as the range followed by the mean in parentheses. The ratio of spore length to thickness (L/T) was also calculated to better describe spore shape.

### *18S* rDNA amplification

For DNA analyses, all kidneys (including those considered uninfected by microscopical examination) were fixed in 400 μl TNES urea buffer [23]. Standard phenol-chloroform DNA extraction was performed after proteinase-K digestion and the obtained DNA was eluted in 50–100 μl DNase/RNase-free PCR grade water. We screened all kidney samples for myxozoan infection by general myxozoan primer combination sets for *18S* rDNA (primer combination of Erib1 + Erib10 followed by second round PCR with MyxGP2F + Act1R; [14, 24]; details in Additional file 2: Table S2, Additional file 3: Table S3, Additional file 4: Table S4). *Sphaerospora 18S* rDNA sequences were obtained by various combinations of general myxozoan, sphaerosporid and species-specific primer sets with specific amplification conditions (Additional file 2: Table S2 and Additional file 4: Table S4). Taq-Purple DNA polymerase (Top-Bio, Prague, Czech Republic) or the more sensitive TITANIUM Taq DNA polymerase (Takara Bio Europe/Clontech, Saint Germain en Laye, France) was used for PCR amplification (Additional file 2: Table S2). PCR products were extracted with the Gel/PCR DNA Fragments Extraction Kit (Geneaid Biotech Ltd., New Taipei City, Taiwan) and sequenced commercially (www.SEQme.eu). All obtained sequences were checked thoroughly for clear chromatograms. In case of mixed sphaerosporid infection, amplicons were cloned into the pDrive vector using the PCR Cloning Kit (Qiagen, Hilden, Germany), and transformed into TOP10 chemically competent *Escherichia coli* cells (Life Technologies, Prague, Czech Republic). Plasmid DNA was isolated by a High Pure Plasmid Isolation Kit (Roche Applied Science, Mannheim, Germany) and three colonies from each PCR product were sequenced commercially. Newly generated sequences were submitted to BLAST (NCBI) for their preliminary identification. Partial sequences were assembled in SeqMan II, DNAStar package v5.05 (DNASTAR Inc., Madison, Wisconsin, USA).

### *18S* rDNA sequence alignment and analyses

Thirty-eight sphaerosporid *18S* rDNA sequences were aligned in MAFFT v7.017 [25] implemented in Geneious v8.0.5 [26] by L-INS-i algorithm, scoring matrix 200PAM/k=2 with gap opening penalty of 1.0 and offset value of 0.1. Due to large insertions in *18S* rDNA variable regions, the alignment was edited manually in Geneious v8.0.5. The complete alignment including extensive species-specific insertions was 4813 bp long (Additional file 5). GC-content was calculated for all newly obtained *18S* rDNA sequences using EditSeq, DNAStar package v5.05 (DNASTAR Inc., Madison, Wisconsin, USA) (Additional file 6: Table S5). Subsequently, nucleotides from extensive long insertions were deleted based on comparison with secondary structure-based alignments [8], resulting in a dataset of 3579 bp which was used for phylogenetic analyses. The distance matrix was produced in Geneious v8.0.5 from the same alignment file after excluding very short partial *18S* rDNA sequences obtained from *Sphaerospora* sp. ex *Gobio gobio* (L.), *Sphaerospora* sp. ex *Rutilus rutilus* (L.), *Sphaerospora elopi* n. sp. and *Sphaerospora hankai* (Additional file 7: Table S6). A more restricted dataset consisting only of *Sphaerospora* spp. obtained from cypriniform hosts (15 taxa, 3768 bp) was produced as it allowed the inclusion of additional informative positions was aligned as described above. Two independent *18S* rDNA datasets were produced to calculate (i) the intraspecific divergence of *Sphaerospora diversa* n. sp. (3 sequences; 3112 bp; Additional file 8: Table S7); and (ii) the interspecific divergence of sphaerosporids obtained from *R. rutilus* (2 sequences; 917 bp; not shown).

### Phylogenetic analyses

Maximum likelihood analysis (ML) was performed using RAxML v7.2.8 [27] implemented in Geneious v8.0.5 with the GTR + Γ model of evolution and 500 bootstrap replicates. jModelTest [28] was used to select the best-fitting model of sequence evolution using corrected Akaike information criterion. Maximum parsimony analysis (MP) was performed in PAUP* v4.b10 [29], using a heuristic search with random taxa addition, the ACCTRAN option and TBR swapping algorithm with bootstrapping analysis for 1000 replicates, gaps were treated as missing data and all characters treated as unordered. Bayesian inference analysis (BI) was performed in MrBayes v3.2.6 [30] implemented in Geneious v8.0.5, using the GTR + Γ model. Posterior probabilities were estimated from 1,000,000 generations by two independent runs of simultaneous Markov Chain Monte Carlo chains with every 100th tree saved. ‘Burn-in’ period was set to 10%; Tracer v1.6 [31] was used to set the length of ‘burn-in’ period.

### Cophylogenetic analyses

Unavailability of mitochondrial data for certain hosts of the 38 sphaerosporids led to replacement of mitogenome sequences with those of closely related species (5 host taxa), while 3 sphaerosporid taxa were withdrawn from the analysis due to unavailability of closely related/congeneric host mitogenome sequences. In addition, 4 partial sphaerosporid *18S* rDNA sequences were excluded due to their short length (details in Additional file 9: Table S8). Hence, for host-parasite cophylogenetic analyses, an alignment of 31 *18S* rDNA *Sphaerospora* (*s.s.*) sequences (3579 bp) was analysed in combination with an alignment of 24 complete host mitogenomes (15 591 bp) available on GenBank (NCBI) (November 2017). Parasite and host ML trees were produced using RAxML v7.2.8 as previously mentioned. Host-parasite cophylogeny was evaluated using an event-based tree topology method, CoRe-PA v0.5.1 [32] without a priori cost assignment, checking 10^4^ cost sets using the simplex method on the quality function. Statistical significance was tested by randomizing host and parasite topologies (10,000 random trees used) under the proportion-to-distinguishable model. As a second method, we determined Global fit based on patristic distances (Geneious v8.0.5, above-mentioned dataset) and independent from tree topologies, in ParaFit [33], implemented in the APE package v3.4 [34] in R v3.2.4 (R Core Team 2013).

## Results

### Infection prevalence in fish

**Fig. 1:**
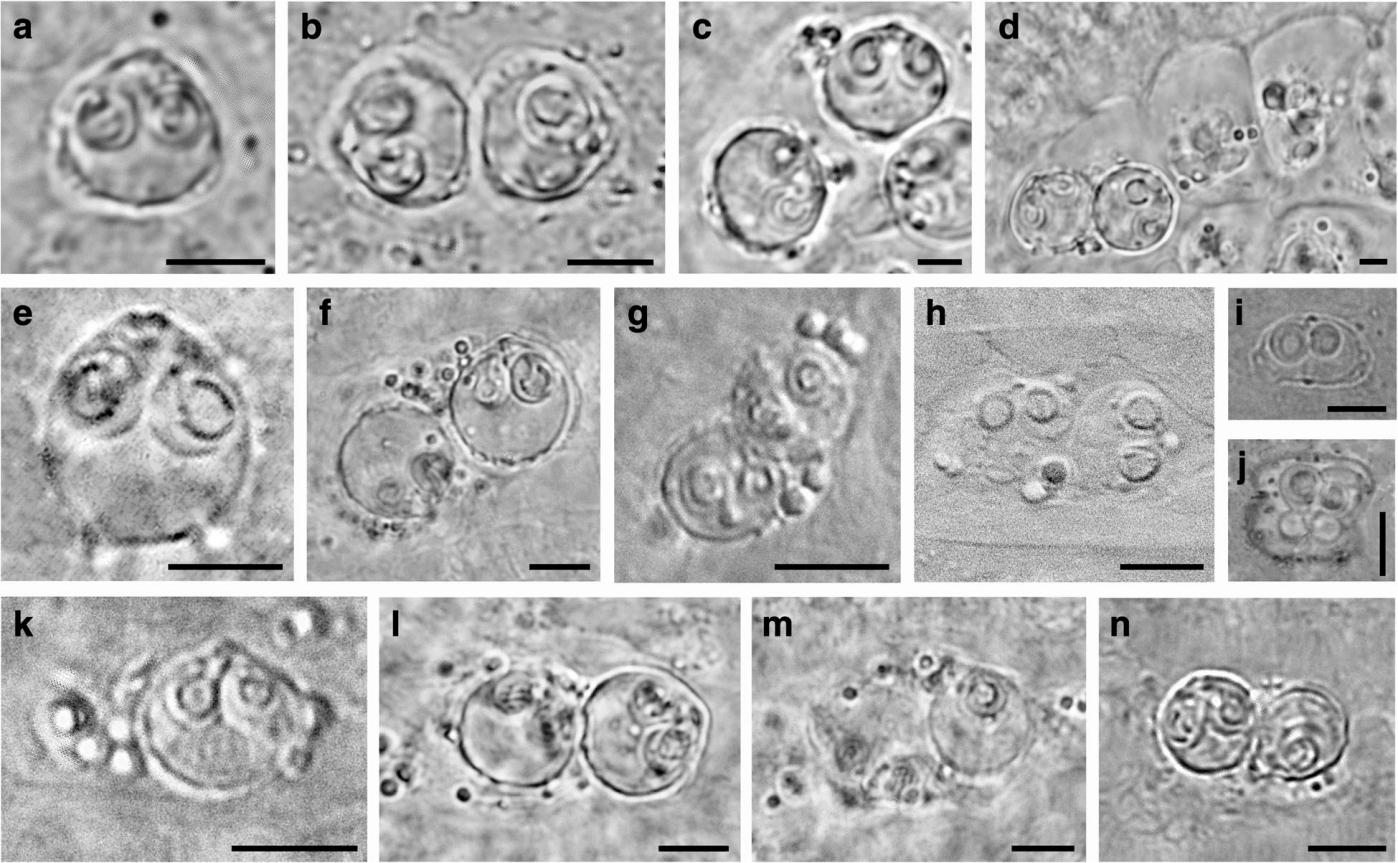
Sphaerosporid pseudoplasmodia and spores within the renal tubules of different fish hosts. **a** Mature spore of *Sphaerospora diminuta*. **b** Disporic pseudoplasmodium of *S. diminuta*. **c** Mature spores of *Sphaerospora abrami* n. sp. **d** Disporic pseudoplasmodium of *S. abrami* n. sp. **e** Mature spore of *Sphaerospora bliccae* n. sp. **f** mature spores of *Sphaerospora dentata* n. sp. **g** Disporic pseudoplasmodium of *Sphaerospora diversa* n. sp. ex *Leuciscus leuciscus*. **h** Disporic pseudoplasmodium of *S. diversa* n. sp. ex *Squalius cephalus*. **i** Mature spore of *Sphaerospora elopi* n. sp. **j** Disporic pseudoplasmodium of *S. elopi* n. sp. **k** Mature spore of *Sphaerospora gutta* n. sp. **l** Disporic pseudoplasmodium of *S. rutili* n. sp. **m** Monosporic pseudoplasmodium of *Sphaerospora rutili* n. sp. **n** Disporic pseudoplasmodium of *Sphaerospora squalii* n. sp*. Scale-bars*: 5 μm

Light microscopic observation determined the presence of early presporogonic stages in the kidney tubules of 101 fish (101/549, 18%) with mature sphaerosporid spores in 16 fish kidney samples (16/549, 3%) (Fig. 1, Tables 1 and 2, Additional file 1: Table S1). PCR screening confirmed “true sphaerosporid” identity of all 16 fish kidneys with sporogonic stages. Moreover, 5 fish kidneys with early presporogonic stages and 5 fish kidneys without any visible infection were PCR-positive for *Sphaerospora* (*s.s.*). Overall prevalence of sphaerosporid infection was very low (26/549, 5%) (Tables 1 and 2, Additional file 1: Table S1). *Abramis brama* (L.), *Rutilus rutilus*, *Scardinius erythrophthalmus* (L.) and *Squalius cephalus* (L.) were found to host multiple sphaerosporid infections. *Sphaerospora diversa* n. sp. was found in three host species from three distinct locations (Table 1). The remaining presporogonic stages (96/549, 18%) belonged to malacosporeans or *Hoferellus* spp. Spores of non-sphaerosporid myxozoans were found in 108 kidney samples (108/549, 20%).

**Table 1:** List of *Sphaerospora* spp. obtained from different fish hosts in this study, including information on PCR, light microscopic detection, locality, the coordinates and *18S* rDNA GenBank accession number

*Sphaerospora* spp.	Host	Locality	Coordinates	Microscopy	PCR positive (%)	*18S* rDNA	
						Sequence length (bp)	GenBank ID
*Sphaerospora abrami* n. sp.	*Abramis brama*	Římov Water Reservoir, CZ	48.8329N, 14.4836E	SPS	1/13 (8)	3100	MG214664
*Sphaerospora* sp.	*Abramis brama*	Lake Balaton, HU	46.8302N, 17.7340E	SPS#	1/1 (100)	3162	KY851765
		Želivka Dam, CZ	49.6743N, 15.1635E	ESPS	1/1 (100)	3162	KY851766
*Sphaerospora bliccae* n. sp.	*Blicca bjoerkna*	Lake Balaton, HU	46.8302N, 17.7340E	SPS	1/1 (100)	3016	KY851767
*Sphaerospora* sp.	*Ctenopharyngodon idella*	Massa Finalese, Modena, IT	44.8505N, 11.2086E	SPS#	1/1 (100)	3306	KY851768
*Sphaerospora dentata* n. sp.	*Scardinius erythrophthalmus*	Želivka Dam, CZ	49.6743N, 15.1635E	SPS	1/1 (100)	3105	MG214666
*Sphaerospora diminuta*	*Lepomis gibbosus*	Jindřiš Fish Farm, CZ	49.1476N, 15.0647E	SPS	1/4 (25)	2665	KY851771
*Sphaerospora diversa* n. sp.	*Leuciscus leuciscus*	River Malše, CZ	48.9095N, 14.4839E	SPS	1/27 (4)	3091	KY863519
		River Oslava, CZ	49.1076N, 16.3505E	ESPS^*@*^	1/2 (50)	3091	KY851772
	*Squalius cephalus*	River Malše, CZ	48.9095N, 14.4839E	ESPS	1/34 (3)	3097	KY851773
	*Leuciscus idus*	River Dyje, CZ	48.6922N, 16.9184E	ESPS^*@*^	1/3 (33)	3049	KY851774
*Sphaerospora elopi* n. sp.	*Elops saurus*	Tidy Island, Florida, USA	27.4426N, 82.6576W	SPS	1/1 (100)	429	KY851769
*Sphaerospora* sp.	*Gobio gobio*	Jindřiš Fish Farm, CZ	49.1476N, 15.0647E	ESPS	1/3 (33)	1196	KY851770
*Sphaerospora gutta* n. sp.	*Scardinius erythrophthalmus*	Jindřiš Fish Farm, CZ	49.1476N, 15.0647E	SPS	1/8 (13)	3306	KY851778
*Sphaerospora* sp.	*Lota lota*	Lake Iseo, Italy	45.7218N, 10.0670E	No visible infection	2/2 (100)	2610	KY851775
							KY863520
*Sphaerospora* sp.	*Rutilus rutilus*	River Dyje, CZ	48.6922N, 16.9184E	No visible infection	1/7 (14)	926	KY851776
*Sphaerospora rutili* n. sp.	*Rutilus rutilus*	Rájský Pond, CZ	49.8294N, 15.4683E	SPS	5/12 (42)	3150	MF347687
*Sphaerospora* sp.	*Sander lucioperca*	Rožmberk Pond, CZ	49.0421N, 14.7757E	No visible infection	1/30 (3)	2847	KY851777
*Sphaerospora* sp.	*Scardinius erythrophthalmus*	Lake Balaton, HU	46.8302N, 17.7340E	SPS#	1/1 (100)	1932	KY851779
*Sphaerospora* sp.	*Silurus glanis*	Lake Milada, CZ	50.6539N, 13.9479E	No visible infection	1/1 (100)	2548	MG214665
*Sphaerospora squalii* n. sp.	*Squalius cephalus*	River Dyje, CZ	48.6922N, 16.9184E	SPS	1/4 (25)	3173	KY851780

*Abbreviations*: *SPS* sporogonic stage containing mature spores, *ESPS* early sporogonic stage (no mature spores), *@* measurement not included due to mixed infection with morphologically similar malacosporean stages, # spore measurements not available, *CZ* Czech Republic, *HU* Hungary, *IT* Italy

**Table 2:** List of *Sphaerospora* spp. identified in the fish hosts in present (bold) and previous studies accompanied by information on spore measurements (in μm) and other important features

Fish host	*Sphaerospora* species	Origin	Organ	Spore	Polar capsule		Pseudoplamodium		Spore surface	Reference
				L × T [L/T ratio]	PL × PW	Polar filament coils	L × W	Development		
*Abramis brama*	*Sphaerospora abrami* n. sp.	CZ	RT	12.2–14.1 × 14.0–15.3 (13.4 ×14.6) [0.9]	Large: 6.6–7.6 × 5.4–7.2 (7.1 × 5.9); small: 5.1–6.8 × 3.8–5.3 (6.0 × 4.7)	2–3	27.4–37.0 × 18.8–28 (32.3 × 22.3)	Disporic, envelope-like structure around sporoblast	Few small posterolateral protuberances on spore valves	Present study
	*Sphaerospora* sp.	CZ, HU	RT	na	na	na	na	na	na	Present study
	*Sphaerospora bramae* El-Matbouli, Hoffmann & Kern, 1995	GR	RT, GM	4.4–5.4 × 4.3–5.4 (5 × 4.9) [1.02]	2.1	na	10	Disporic	Posterior end with 2 fine ridges on each spore valve	[39]
	*Sphaerospora masovica* Cohn, 1902	CN	GB, IN	8 × 8 [1.0]	na	na	10–38	Di- and polysporic	Smooth valve surface	[40]
	*Sphaerospora* sp.	CZ, SK	RT	na	na	na	na	na	na	[41]
	*Sphaerospora* sp.	HU	RT	na	na	na	na	na	na	[42]
*Blicca bjoerkna*	*Sphaerospora bliccae* n. sp.	HU	RT	9.7–11.1 × 9.3–10.1 (10.40 × 9.60 [1.1]	3.9–4.7 × 3.8–4.2 (4.3 × 3.9)	4–5	na	na	Smooth valve surface, apical end split	Present study
*Ctenopharygodon idella*	*Sphaerospora* sp.	IT	RT	na	na	na	na	na	na	Present study
	*Sphaerospora carassii* Kudo, 1919	JP	GL	8–13 [0.62]	4–5	na	10–20	Disporic	Smooth valve surface	[40, 59]
	*Sphaerospora ctenopharyngodoni* Chen, 1998	CH	GL	14.7 × 12.0 [1.23]	5.9 × 4.8	4–5	na	Mono- and disporic	Smooth valve surface	[75]
	*Sphaerospora kwangtungensis* Chen, 1998	CH	GL	9.5 × 7.6 [1.25]	3.1 × 2.9	3–4	na	na	10–12 longitudinal striation	[75]
	*Sphaerospora* sp.	HU	KD	na	na	na	na	na	na	[42]
*Elops saurus*	*Sphaerospora elopi* n. sp.	USA	RT	5.8–6.6 × 8.9–10.5 (6.4 × 10.0) [0.64]	large: 3.2–4.0 × 2.7–3.3 (3.5 × 3.0); small: 3.0–3.9 × 2.6–3.2 (3.0 × 2.9)	na	26.3–34.6 × 22.3–26.0 (30.5 × 24.2)	Disporic	Broad posterolateral bulges on each valve	Present study
*Gobio gobio*	*Sphaerospora* sp.	CZ	RT	na	2.2–2.4 × 2.0–2.1 (2.3 × 2.1)^b^	na	8.4–13.2 × 3.3–10.0 (10.2 × 6.3)	Monosporic	na	Present study
	*Sphaerospora gobionis* Lom, Pavlovskova & Dyková, 1985	SK	RT	6.5–8.0 × 6.4–7.7 (7.1 × 6.9) [1.03]	3.2–3.7 × 2.8–3.0 (3.4 × 2.9)	3–4	15	Disporic	Valve surface uneven	[41]
	*Sphaerospora* sp.	HU	BL, KD	na	na	na	na	na	na	[42]
*Lepomis gibbosus*	*Sphaerospora diminuta* Li & Desser, 1985	CN	RT	5.0–8.5 (7.5) [1.0]	2–3 (2.5)	3–4	na	Disporic	3–4 (4–6 in [33]) striations from apical end toward posterolateral bulges on each valve	[35]
		CN	RT, BL	6.7–7.5 × 6.7–8 (7.0 × 7.6) [0.92]	2.5–3.0 (2.9)	4–5	Up to 15			[36]
		CZ	RT	7.3–8.7 × 7.6–9.7 (8.1 × 8.6) [0.94]	3.0–4.2 × 2.9–4.4 (3.7 × 3.4)	3–4	13.6–16.6 × 6.6–11.2 (15.0 × 8.8)			Present study
	*Sphaerospora ovophila* Xiao & Desser, 1997	CN	OV	7.2–8.4 × 7.4–8.2 (8.2 × 7.9) [1.03]	2.7–3.2 × 2.6–3.1 (3.0 × 2.8)	6–7	12.5	Monosporic	5–6 ridges on surface of posterior end of each valve	[37]
*Leuciscus idus*	*Sphaerospora diversa* n. sp.	CZ	RT	na	na	na	na	na	na	Present study
*Leuciscus leuciscus*	*Sphaerospora diversa* n. sp.	CZ	RT	5.9–6.0 × 6.4–6.6 (6.0 × 6.5) [0.92]	3.0 × 2.2–2.6 (3.0 × 2.4)	na	na	Disporic	Smooth valve surface	Present study
	“Sphaerospora leuciscusi” (*nomen nudum*) of Longshaw (2004) [44]	EN	RT	5.11–6.01 × 5.40–6.72 (5.56 × 5.87) [0.95]	1.97–3.13 (2.35)	3–4	14	Monosporic	Smooth valve surface	[44]
	*Sphaerospora rota* Zaika, 1961^a^	RS	RT, UB, UR	8.4–11.0 × 9.1–9.8 [na]	4.2–5.6 × 2.8–4.2	na	12–14	na	3 small lateral protuberances, one posterior protrusion on each valve	[59]
*Lota lota*	*Sphaerospora* sp.	IT	KD	na	na	na	na	na	na	Present study
	*Sphaerospora cristata* Shulman, 1962	RS	UB, UR	9–10 × 8.3–10 [na]	3.5–4.0 × 2.5–3.0	na	na	na	3 posterolateral denticles on each spore valve	[59]
*Rutilus rutilus*	*Sphaerospora rutili* n. sp.	CZ	RT	8.3–9.8 × 8.8–9.3 (8.8 × 8.9) [0.99]	3.2–4.2 × 2.7–3.5 (3.7 × 3.2)	3–4	13.7–24.0 × 7.2–17.6 (19.0 × 10.4)	Mono- and disporic	3–4 small humps posterolaterally on each valve	Present study
	*Sphaerospora* sp.	CZ	RT	na	na	na	na	na	na	Present study
	*Sphaerospora carassii* Kudo, 1919	RS, HU, JP	GL	8–13 [na]	4–5	na	10–20	Disporic	Smooth valve surface	[40, 59]
	*Sphaerospora minima* Kaschkovsky, 1974	RS	UB	6.0–7.7 × 5.4–6.0 [na]	2.8–3.0 × 2.3–2.8	na	na	na	Posterior end with spine-like ornamentations arranged in 3 lines	[49]
	“Sphaerospora ousei” (*nomen nudum*) of Longshaw (2004) [44]	EN	RT	7.56–9.12 × 7.54–8.89 (8.46 × 8.21) [1.03]	2.60–3.89 (3.22)	3–4	18–21 × 10–11	Mono- and disporic	Smooth valve surface	[44]
	*Sphaerospora poljanskii* Kulemina, 1969	RS	RT	9.5–10.0 × 9.0–10.0 [na]	3.0–4.0 × 2.5–3.0	na	na	na	Split at apical end, 2 triangular posterolateral projections	[48]
	*Sphaerospora* sp. 1	CZ	RT	8.5 × 8.2 [1.04]	3.5 × 2.9	3	na	na	na	[41]
	*Sphaerospora* sp. 2	CZ	RT	7 × 7 [1.0]	3.3 × 2.9	5	14	na	Smooth valve surface	[41]
	*Sphaerospora* sp.	CZ	RT	na	na	5	na	na	Ornate posterolateral valve end	[50]
*Sander lucioperca*	*Sphaerospora* sp.	CZ	RT	na	na	na	na	na	na	Present study
	*Sphaerospora danubialis* Molnár, 1991	HU	RT	9.5–11.0 × 8.5–10.0 (10.1 × 8.7) [1.16]	4.0–4.8 (4.4)	5–6	9–14 × 12–19	Disporic	Rounded triangular shape, protruding tubercules	[60]
	*Sphaerospora luciopercae* Moshu, 1992	ML, UK	OV	7.2–9.6 × 5.0–7.0 (7.6 × 6.2) [1.23]	3–4 × 2.5–3.6 (3.8 × 3.0 )	3–4	9.0–18.0 × 7.5–10.0	Polysporic (4 or more)	Scale-shaped elevation at posterior end	[80]
*Scardinius erythrophthalmus*	*Sphaerospora dentata* n. sp.	CZ	RT	9.4–9.8 × 9.4–9.6 (9.7 × 9.4) [1.03]	3.9–4.2 × 3.2–3.6 (4.0 × 3.5)	4–5	17.6–22.1 × 10.5–12.5 (20.1 × 11.5)	Disporic	Multiple subtle posterolateral protuberances, 3 ridges at posterior end of spore valve	Present study
	*Sphaerospora gutta* n. sp.	CZ	RT	6.2–6.6 × 6.3–6.7 (6.4 × 6.5) [0.98]	2.3–2.6 × 2.2–2.5 (2.5 × 2.3)	4–5	5.3–18.8 × 4.6–12.4 (11.1 × 6.9)	Mono- and disporic	Smooth valve surface	Present study
	*Sphaerospora* sp.	HU	RT	na	na	na	na	na	na	Present study
	*Sphaerospora scardinii* El-Matbouli & Hoffmann, 1992	GR	RT, UR	5.97–7.25 × 5.31–6.30 (6.01 × 5.79) [1.04]	1.8–2.93 × 1.82–2.24 (2.26 × 2.14)	4–5	17.49	Mono- and disporic	3–4 fine ridges at posterior end	[43]
	*Sphaerospora* sp.	HU	BL, KD	na	na	na	na	na	na	[42]
	*Sphaerospora* sp.	HU	KD	na	na	na	na	na	na	[46]
	*Sphaerospora* sp.	IR	KD	na	na	na	na	na	na	[47]
*Silurus glanis*	*Sphaerospora* sp.	CZ	RT	na	na	na	na	na	na	Present study
	*Sphaerospora siluri* Molnár, 1993	HU	RT	6.6–6.9 × 6.7–6.9 (6.8 × 6.8)	2.8–3.4 (3.1)	5–6		Monosporic	Triangular spore shape with two lateral ear-like protrusions	[97]
*Squalius cephalus*	*Sphaerospora diversa* n. sp.	CZ	RT	6.3–6.7 × 7.0–7.9 (6.5 × 7.5) [0.87]^b^	2.3–2.7 × 2.3–2.5 (2.5 × 2.4)^b^	na	8.0–14.9 × 5.4–8.3 (12.1 × 7.0)	Disporic	na	Present study
	*Sphaerospora squalii* n. sp.	CZ	RT	6.1–7.1 × 6.2–7.0 (6.5 × 6.5) [1.0]	3.0–3.4 × 2.7–3.0 (3.2 × 2.8)	3–4	8.2–15.1 × 7.2–9.8 (10.1 × 8.3)	Mono- and disporic	Projected posterior end with subtle posterolateral protuberances	Present study
	*Sphaerospora* sp.	CZ, SK	RT	na	na	na	na	na	na	[41]

^a^From *Leuciscus leuciscus baicalensis*

^b^Measurements from immature spores

*Abbreviations*: *L* length, *W* width, *T* thickness, *PF* polar filament, *BL* blood, *GB* gall-bladder, *GL* gills, *GM* glomeruli, *IN* intestine, *KD* kidney, *OV* ovary, *RT* renal tubules, *UB* urinary bladder, *UR* ureter, *CH* China, *CN* Canada, *CZ* Czech Republic, *SK* Slovakia, *EN* England, Great Britain, *GR* Germany, *HU* Hungary, *IR* Ireland, *IT* Italy, *JP* Japan, *ML* Moldova, *RU* Russia, *UN* Ukraine, *na* data not available

### Sphaerosporid species diversity and descriptions

Based on strongly divergent sequences in the variable sections of the *18S* rDNA gene region, 17 new *18S* rDNA sequences of were obtained from the fish examined in the present study. Based on morphology, *Sphaerospora diminuta* Li & Desser, 1985 was identified and redescribed from *Lepomis gibbosus* (L.) caught in the Czech Republic (Fig. 1a and b, Table 2). Eight taxa are new species for which we provide morphological and molecular data. The lack of microscopically detectable mature and immature spores or the presence of mixed sphaerosporid infections prevent us from identifying the other eight sphaerosporids detected by *18S* rDNA sequencing (Tables 1 and 2, Additional file 1: Table S1) from *A. brama*, *Ctenopharyngodon idella* (Valenciennes), *Gobio gobio*, *Lota lota* (L.), *Sander lucioperca* (L.), *S. erythrophthalmus*, *Silurus glanis* L. and *R. rutilus*. These may represent new species or species that have been previously described or recorded in these hosts (Table 2).

### Descriptions of species of *Sphaerospora*

- **Phylum Cnidaria Hatschek, 1888**
- **Unranked subphylum Myxozoa Grassé, 1970**
- **Class Myxosporea Bütschli, 1881**
- **Order Bivalvulida Schulman, 1959**
- **Suborder Variisporina Lom & Noble, 1984**
- **Family Sphaerosporidae Davis, 1917**
- **Genus** ***Sphaerospora*** **Thélohan, 1892**

### *Sphaerospora diminuta* Li & Desser 1985

- ***Type-host*:** *Lepomis gibbosus* (L.) (Centrarchiformes: Centrarchidae), pumpkinseed.
- ***Type-locality*:** Lake Sasajewun, Ontario [35, 36].
- ***Other locality*:** Jindřiš fish farm (49.1476N, 15.0647E), Czech Republic (present study).
- ***Voucher material*:** Series of photomicrographs, and a representative DNA sample was deposited at the Protistological Collection of the Institute of Parasitology, Biology Centre, Czech Academy of Sciences, České Budějovice (accession number IPCAS Prot 47).
- ***Site in host*:** Lumen of renal tubules and ureters.
- ***Prevalence***: 20% (2/10) [35], 18% (7/40) [36] and 25% (1/4) (present study).
- ***Representative DNA sequence*:** A partial *18S* rDNA sequence of 2665 bp was deposited in the GenBank database under the accession number KY851771.

### Redescription

**Fig. 2:**
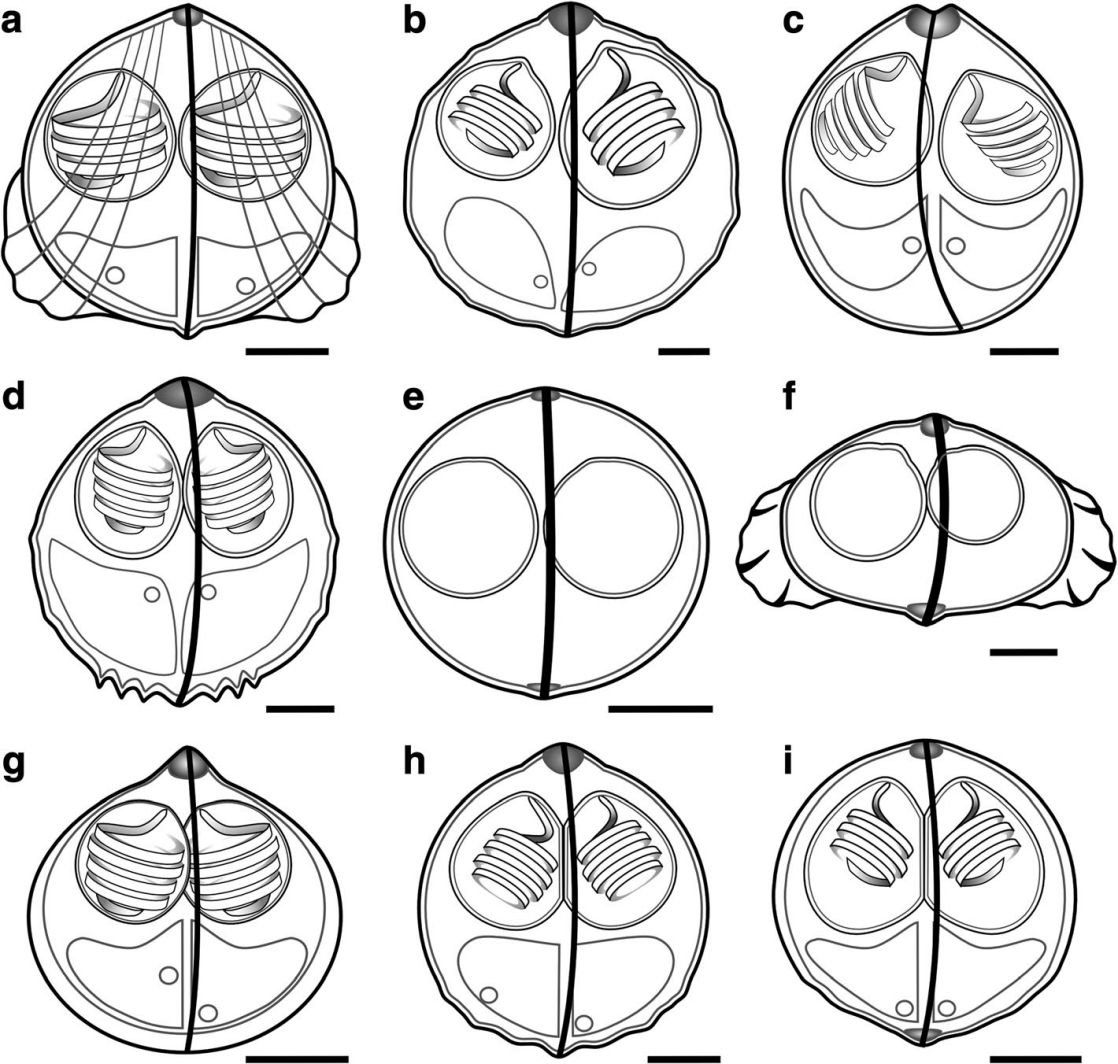
Line drawings of the *Sphaerospora* spp. spores obtained from renal tubules of different fish hosts. **a** *Sphaerospora diminuta*. **b** *Sphaerospora abrami* n. sp. **c** *Sphaerospora bliccae* n. sp. **d** *Sphaerospora dentata* n. sp. **e** *Sphaerospora diversa* n. sp. ex *Leuciscus leuciscus.* **f** *Sphaerospora elopi* n. sp. **g** *Sphaerospora gutta* n. sp. **h** *Sphaerospora rutili* n. sp. **i** *Sphaerospora squalii* n. sp*. Scale-bars*: 2 μm

***Spore.*** Mature spores subspherical with pointed apical end, measuring 7.3–8.7 × 7.6–9.7 (8.1 × 8.6) (L × T, *n* = 6) (Figs. 1a and 2a). Spores with 2 equally-sized subspherical polar capsules, 3.0–4.2 × 2.9–4.4 (3.7 × 3.4) (PL × PW, *n* = 12). Polar filaments with 3–4 (*n* = 6) coils per polar capsule. Spore valves with 3–4 longitudinal striations, with posterolateral bulges on each valve; sutural line straight, prominent, with sutural ridge protruding slightly at spore apex. Sporoplasms 2, uninucleate.

***Pseudoplasmodium.*** Disporic pseudoplasmodia measuring 13.6–16.6 × 6.6–11.2 (15.0 × 8.8) (*n* = 9), with numerous refractile granules (Fig. 1b).

### Remarks

Spore measurements, spore surface ornamentation, development of disporic pseudoplasmodia, host tissue localization and the number of polar filament coils match the original description of *S. diminuta* [35] and its later report [36] though motility of the pseudoplasmodia and higher number of spore surface striations (4–6 *vs* 3–4) reported by Lom et al. [36] were not observed in the present study (Table 2). In addition, extrasporogonic blood stages were observed by Lom et al. [36]. For the first time, we are providing *18S* rDNA sequence data of this species. Another species, *Sphaerospora ovophila* Xiao & Desser, 1997 from the ovary of *L. gibbosus* significantly differs by spore measurements, number of polar filament coils, L/T ratio (Table 2), formation of monosporic pseudoplasmodia and tissue localization. Additionally, ornamental folds (pits) in *S. ovophila* occur only on the posterior end of the spores which contrasts ridges overarching the whole spore and the additional presence of posterolateral bulges in *S. diminuta* (Table 2) [37].

### *Sphaerospora abrami* n. sp.

- ***Type-host*:** *Abramis brama* (L.) (Cypriniformes: Leuciscidae), common bream.
- ***Type-locality***: Římov Water Reservoir (48.8329N, 14.4836E), Czech Republic.
- ***Type-material*:** Hapantotypes: series of phototypes, deposited together with a representative DNA sample in the Protistological Collection of the Institute of Parasitology, Biology Centre, Czech Academy of Sciences, České Budějovice (accession number IPCAS Prot 44).
- ***Site in host*:** Lumen of renal tubules.
- ***Prevalence*:** 8% (1/13).
  ***Representative DNA sequence:*** A partial *18S* rDNA sequence of 3100 bp was deposited in the GenBank database under the accession number MG214664.
- ***ZooBank registration*:** To comply with the regulations set out in article 8.5 of the amended 2012 version of the *International Code of Zoological Nomenclature* (ICZN) [38], details of the new species have been submitted to ZooBank. The Life Science Identifier (LSID) of the article is urn:lsid:zoobank.org:pub:CFA20253-62B5-4BC0-8F30-2C054C1DB0D9. The LSID for the new name *Sphaerospora abrami* is urn:lsid:zoobank.org:act:F2B82DC9-697E-48C5-8E99-56C8BFA414BB.
- ***Etymology*:** The species epithet “*abrami*” is derived from the type-host species name *Abramis brama*.

### Description

***Spore.*** Mature spores subspherical, with pointed apical end, measuring 12.2–14.1 × 14.0–15.3 (13.4 × 14.6) (L × T, *n* = 6) (Figs. 1c and 2b). Spores with 2 unequally-sized subspherical polar capsules; larger capsule measuring 6.6–7.6 × 5.4–7.2 (7.1 × 5.9) (PL × PW, *n* = 6); smaller capsule measuring 5.1–6.8 × 3.8–5.3 (6.0 × 4.7) (PL × PW, *n* = 6). Polar filaments with 2–3 (*n* = 8) coils per polar capsule. Spore surface smooth with few small posterolateral protuberances; sutural line straight, prominent, with sutural ridge protruding slightly at anterior end. Sporoplasms 2, uninucleate.

***Pseudoplasmodium.*** Disporic pseudoplasmodia measuring 27.4–37.0 × 18.8–28.0 (32.3 × 22.3) (*n* = 11), with numerous refractile granules (Fig. 1d).

### Remarks

There are two sphaerosporids described from *A. brama*, *Sphaerospora bramae* El-Matbouli, Hoffmann & Kern, 1995, infecting the renal tubules was described from Germany [39] and *Sphaerospora masovica* Cohn, 1902, infecting the gall-bladder and intestine from Canada [40]. Both species are much smaller than the present species, nevertheless, posterior ridges are present on both, *S. bramae* and *S. abrami* n. sp. (Table 2). Moreover, pseudoplasmodia of two sphaerosporids (without mature spores) were reported from the renal tubules of the same fish host [41, 42]. In the present study, another species, *Sphaerospora* sp. ex *A. brama* (without morphological data) differs by over 11% from the *18S* rDNA of *S. abrami* n. sp., which confirms their distinct species status. Unavailability of molecular data from previously reported species impede further comparisons with the new species.

#### *Sphaerospora bliccae* n. sp.

- ***Type-host*:** *Blicca bjoerkna* (L.) (Cypriniformes: Leuciscidae), white bream.
- ***Type-locality*:** Lake Balaton (46.8302N, 17.7340E), Hungary.
- ***Type-material*:** Hapantotypes: series of phototypes, deposited together with a representative DNA sample in the Protistological Collection of the Institute of Parasitology, Biology Centre, Czech Academy of Sciences, České Budějovice (accession number IPCAS Prot 45).
- ***Site in host*:** Lumen of renal tubules.
- ***Prevalence*:** 100% (1/1).
- ***Representative DNA sequence*:** A partial *18S* rDNA sequence of 3016 bp was deposited in the GenBank database under the accession number KY851767.
- ***ZooBank registration*:** To comply with the regulations set out in article 8.5 of the amended 2012 version of the *International Code of Zoological Nomenclature* (ICZN) [38], details of the new species have been submitted to ZooBank. The Life Science Identifier (LSID) of the article is urn:lsid:zoobank.org:pub:CFA20253-62B5-4BC0-8F30-2C054C1DB0D9. The LSID for the new name *Sphaerospora bliccae* is urn:lsid:zoobank.org:act:49618F86-3AFA-44EA-9F91-5EAB76DBE12C.
- ***Etymology*:** The species epithet “*bliccae*” is derived from the type-host species name *Blicca bjoerkna*.

### Description

***Spore.*** Mature spores subspherical, measuring 9.7–11.1 × 9.3–10.1 (10.4 ± 0.4 × 9.6 ± 0.2) (L × T, *n* = 25) (Figs. 1e and 2c). Each spore with 2 equally-sized subspherical polar capsules, measuring 3.9–4.7 × 3.8–4.2 (4.3 ± 0.2 × 3.9 ± 0.2) (PL × PW, *n* = 37). Polar filaments with 4–5 (*n* = 9) coils. Spore valves smooth, with slightly curved, pronounced sutural line, moderate apical protrusion with marked dent, devoid of any other ornamentation. Sporoplasms 2, uninucleate.

### Remarks

Sphaerosporid blood stages and spores were reported from the kidney of *B. bjoerkna* [42] but lacked morphological or molecular data for comparison.

### *Sphaerospora dentata* n. sp.

- ***Type-host*:** *Scardinius erythrophthalmus* (L.) (Cypriniformes: Leuciscidae), common rudd.
- ***Type-locality*:** Želivka Dam (49.6743N, 15.1635E), Czech Republic.
- ***Type-material*** Hapantotypes: series of phototypes, deposited together with a representative DNA sample in the Protistological Collection of the Institute of Parasitology, Biology Centre, Czech Academy of Sciences, České Budějovice (accession number IPCAS Prot 46).
- ***Site in host*:** Lumen of renal tubules.
- ***Prevalence*:** 100% (1/1).
  ***Representative DNA sequence*:** A partial *18S* rDNA sequence of 3105 bp was deposited in the GenBank database under the accession number MG214666.
- ***ZooBank registration*:** To comply with the regulations set out in article 8.5 of the amended 2012 version of the *International Code of Zoological Nomenclature* (ICZN) [38], details of the new species have been submitted to ZooBank. The Life Science Identifier (LSID) of the article is urn:lsid:zoobank.org:pub:CFA20253-62B5-4BC0-8F30-2C054C1DB0D9. The LSID for the new name *Sphaerospora dentata* is urn:lsid:zoobank.org:act:319CE294-0575-4915-8B7D-95E956C72D44.
  ***Etymology*:** The species epithet “*dentata*” is referred to the tooth-like pointed ridges of the posterior spore valve surface.

### Description

***Spore.*** Mature spores subspherical, with pointed apical end, measuring 9.4–9.8 × 9.4–9.6 (9.7 ± 0.3 × 9.4 ± 0.4) (L × T, *n* = 18) (Figs. 1f and 2d). Spores with 2 equally-sized subspherical polar capsules measuring 3.9–4.2 × 3.2–3.6 (4.0 ± 0.2 × 3.5 ± 0.3) (PL × PW, *n* = 36). Polar filaments with 4–5 (*n* = 20) coils per polar capsule. Spore surface with multiple subtle posterolateral protuberances and 3 prominent pointed ridges on posterior end of each spore valve; sutural line straight, prominent, with sutural ridge protruding slightly at anterior pole. Sporoplasms 2, uninucleate.

***Pseudoplasmodium.*** Disporic pseudoplasmodia measuring 17.6–22.1 × 10.5–12.5 (20.1 × 11.5) (*n* = 11), with numerous refractile granules.

### Remarks

The spore measurements and the development of disporic pseudoplasmodia differentiate *S. dentata* n. sp. from *Sphaerospora scardinii* El-Matbouli & Hoffmann, 1992 [43], described from same host. Both species have ridges at the posterior end of the mature spores (Table 2). Further details are provided in *Sphaerospora gutta* n. sp. Remarks section.

### *Sphaerospora diversa* n. sp.

- ***Type-host*:** *Leuciscus leuciscus* (L.) (Cypriniformes: Leuciscidae), common dace.
- ***Other hosts*:** *Leuciscus idus* (L.) (Cypriniformes: Leuciscidae), ide; and *Squalius cephalus* (Cypriniformes: Leuciscidae), European chub.
- ***Type-locality*:** River Malše (48.9095N, 14.4839E), Czech Republic.
- ***Other localities*:** River Oslava (49.1076N, 16.3505E) and River Dyje (48.6922N, 16.9184E), Czech Republic.
- ***Type-material*:** Hapantotypes: series of phototypes, deposited together with a representative DNA sample in the Protistological Collection of the Institute of Parasitology, Biology Centre, Czech Academy of Sciences, České Budějovice (accession number IPCAS Prot 48).
- ***Site in host*:** Lumen of renal tubules.
- ***Prevalence*:** 3–50% (details in Table 1).
- ***Representative DNA sequences*:** Identical partial *18S* rDNA sequences of 3091 bp obtained from *L. leuciscus* from two different sites in the Czech Republic were deposited in the GenBank database under accession numbers KY851772 and KY863519. Partial *18S* rDNA sequences from *L. idus* (3049 bp) and *S. cephalus* (3097 bp) were deposited in the GenBank database under accession numbers KY851774 and KY851773, respectively.
- ***ZooBank registration*:** To comply with the regulations set out in article 8.5 of the amended 2012 version of the *International Code of Zoological Nomenclature* (ICZN) [38], details of the new species have been submitted to ZooBank. The Life Science Identifier (LSID) of the article is urn:lsid:zoobank.org:pub:CFA20253-62B5-4BC0-8F30-2C054C1DB0D9. The LSID for the new name *Sphaerospora diversa* is urn:lsid:zoobank.org:act:34631CAD-DC47-405C-B3F7-4D2297A21FE9.
- ***Etymology*:** The species epithet “*diversa*” is referred to a widened host specificity (three leuciscinid host species) of the species.

### Description

***Spore.*** Mature spores from *Leuciscus leuciscus* measuring 5.9–6.0 × 6.4–6.6 (6.0 × 6.5) (L × T, *n* = 2) with 2 equally-sized subspherical polar capsules measuring 3.0 × 2.2–2.6 (3.0 × 2.4) (PL × PW, *n* = 2) (Figs. 1g and 2e). Immature spores from *S. cephalus* measuring 6.3–6.7 × 7.0–7.9 (6.5 × 7.5) (L × T, *n* = 2), with 2 equally-sized subspherical polar capsules, measuring 2.3–2.7 × 2.3–2.5 (2.5 × 2.4) (PL × PW, *n* = 5) (Fig. 1h). Spore surface smooth, without ornamentation; sutural line straight, thick, prominent, slightly protruding at anterior spore end.

***Pseudoplasmodium.*** Disporic pseudoplasmodia from *S. cephalus* measuring 8.0–14.9 × 5.4–8.3 (12.1 × 7.0) (*n* = 11), with numerous refractile granules.

### Remarks

The low *18S* rDNA sequence divergence (0.29–0.89% over 3,112 bp; Additional file 8: Table S7) amongst the isolates of *S. diversa* n. sp.**,** similar spore measurements and L/T ratios < 1 (Table 2) confirm the conspecificity of these three isolates. So far, only “Sphaerospora leuciscusi” (*nomen nudum*) of Longshaw (2004) [44] has been described from the kidney of *L. leuciscus* [44] and *Sphaerospora rota* Zaika, 1961 has been reported from the kidney of *Leuciscus leuciscus baicalensis*, a subspecies of dace in Lake Baikal [45]. The present species has similar spore and polar capsule measurements as “S. leuciscusi” although it develops exclusively in monosporic pseudoplasmodia and has different L/T ratio (Table 2). Spores of *S. diversa* n. sp. are significantly smaller than those of *S. rota* (Table 2), which also differs by a strongly protruding sutural edge, three small lateral protuberances and a prominent ridge on the posterior spore pole. *Sphaerospora rota* may represent a species complex as it was also reported from distantly related cypriniform fish *Cobitis taenia* L. and salmoniform fish *Brachymystax lenok* (Pallas) [45]. Molecular data from both *S. leuciscusi* and *S. rota* are not available for comparison with our reports. No sphaerosporid was previously described from *S. cephalus* and *L. idus*. Pseudoplasmodia of an undescribed sphaerosporid were reported in the renal tubules of *S. cephalus* [41], without morphological or DNA sequence data for species comparison.

### *Sphaerospora elopi* n. sp.

- ***Type-host*:** *Elops saurus* L. (Elopiformes: Elopidae), ladyfish.
- ***Type-locality*:** Tidy Island (27.4426N, 82.6576W), Florida, USA.
- ***Type-material*:** Hapantotypes: series of phototypes, deposited together with a representative DNA sample in the Protistological Collection of the Institute of Parasitology, Biology Centre, Czech Academy of Sciences, České Budějovice (accession number IPCAS Prot 49).
- ***Site in host*:** Lumen of renal tubules.
  ***Prevalence*:** 100% (1/1).
- ***Representative DNA sequence*:** A partial *18S* rDNA sequence of 429 bp was deposited in the GenBank database under the accession number KY851769.
- ***ZooBank registration*:** To comply with the regulations set out in article 8.5 of the amended 2012 version of the *International Code of Zoological Nomenclature* (ICZN) [38], details of the new species have been submitted to ZooBank. The Life Science Identifier (LSID) of the article is urn:lsid:zoobank.org:pub:CFA20253-62B5-4BC0-8F30-2C054C1DB0D9. The LSID for the new name *Sphaerospora elopi* is urn:lsid:zoobank.org:act:D04A2079-3D73-46C4-87F6-07DA80C174CC.
- ***Etymology*:** The species epithet “*elopi*” is derived from the type-host species name *Elops saurus*.

### Description

***Spore.*** Mature spores thicker than wide,, measuring 5.8–6.6 × 8.9–10.5 (6.4 × 10.0) (L × T, *n* = 9) (Figs. 1i and 2f). Polar capsules 2, unequally-sized, subspherical; larger capsule measuring 3.2–4.0 × 2.7–3.3 (3.5 × 3.0) (PL × PW, *n* = 9); smaller capsule measuring 3.0–3.9 × 2.6–3.2 (3.0 × 2.9) (PL × PW, *n* = 9). Broad posterolateral bulges present on spore valves of mature spores;.sutural line straight, thick, prominent, slightly protruding at both anterior and posterior spore end.

***Pseudoplasmodium.*** Pseudoplasmodia diasporic, measuring 26.3–34.6 × 22.3–26.0 (30.5 × 24.2) (*n* = 2) (Fig. 1j).

### Remark

*Elops saurus* or other elopid fishes were not previously reported to harbour sphaerosporids.

### *Sphaerospora gutta* n. sp.

- ***Type-host*:** *Scardinius erythrophthalmus* (L.) (Cypriniformes: Leuciscidae), common rudd.
- ***Type-locality*:** Jindřiš Fish Farm (49.1476N, 15.0647E), Czech Republic.
- ***Type-material*:** Hapantotypes: series of phototypes, deposited together with a representative DNA sample in the Protistological Collection of the Institute of Parasitology, Biology Centre, Czech Academy of Sciences, České Budějovice (accession number IPCAS Prot 50).
- ***Site in host*:** Lumen of renal tubules.
  ***Prevalence*:** 13% (1/8).
- ***Representative DNA sequence*:** A partial *18S* rDNA sequence of 3306 bp was deposited in the GenBank database under the accession number KY851778.
- ***ZooBank registration*:** To comply with the regulations set out in article 8.5 of the amended 2012 version of the *International Code of Zoological Nomenclature* (ICZN) [38], details of the new species have been submitted to ZooBank. The Life Science Identifier (LSID) of the article is urn:lsid:zoobank.org:pub:CFA20253-62B5-4BC0-8F30-2C054C1DB0D9. The LSID for the new name *Sphaerospora gutta* is urn:lsid:zoobank.org:act:165124A9-3CA4-471F-BD02-44D97AE696D8.
- ***Etymology*:** The species epithet “*ghutta*” is referred to the drop-like mature spore shape.

### Description

***Spore.*** Spores drop-shaped, subspherical, measuring 6.2–6.6 × 6.3–6.7 (6.4 × 6.5) (L × T, *n* = 2) (Figs. 1k and 2g). Spores with 2 equally-sized subspherical polar capsules measuring 2.3–2.6 × 2.2–2.5 (2.5 × 2.3) (PL × PW, *n* = 4). Polar filaments with 4–5 (*n* = 4) coils per polar capsule. Spore surface smooth, sutural line straight, with prominent sutural ridge protruding at anterior spore end. Sporoplasms 2, uninucleate.

***Pseudoplasmodium.*** Pseudoplasmodia mostly monosporic and rarely diasporic, measuring 5.3–18.8 × 4.6–12.4 (11.1 ± 3.7 × 6.9 ± 1.6) (*n* = 22), with numerous refractile granules.

### Remarks

*Sphaerospora. gutta* n. sp. is similar to *S. scardinii* described from the same host when comparing spore measurements, development in mono- and disporic pseudoplasmodia, within-host localization and the number of polar filament coils (Table 2) [43]. However, fine ridges found at the posterior end of *S. scardinii* were never observed in our samples. For similar reasons, the present species differs significantly from *S. dentata* n. sp. (Table 2). *18S* rDNA data confirm the distinct status of these two new species and another species *Sphaerospora* sp. ex *S. erythrophthalmus*, which lacks morphological data (see below and Additional file 7: Table S6). Absence of *18S* rDNA data from *S. scardinii* impedes comparison with this species. Undescribed *Sphaerospora* spp. were reported in the blood and kidney of *S. erythrophthalmus* but the lack of spore details and molecular data impede further comparison [42, 46, 47].

### *Sphaerospora rutili* n. sp.

- ***Type-host*:** *Rutilus rutilus* (L.) (Cypriniformes: Leuciscidae), common roach.
- ***Type-locality*:** Rájský Pond (49.8294N, 15.4683E), Czech Republic.
- ***Type-material*:** Hapantotypes: series of phototypes, deposited together with a representative DNA sample in the Protistological Collection of the Institute of Parasitology, Biology Centre, Czech Academy of Sciences, České Budějovice (accession number IPCAS Prot 51).
- ***Site in host*:** Lumen of renal tubules.
  ***Prevalence*:** 42% (5/12).
- ***Representative DNA sequence*:** A partial *18S* rDNA sequence of 3150 bp was deposited in the GenBank database under the accession number MF347687.
- ***ZooBank registration*:** To comply with the regulations set out in article 8.5 of the amended 2012 version of the *International Code of Zoological Nomenclature* (ICZN) [38], details of the new species have been submitted to ZooBank. The Life Science Identifier (LSID) of the article is urn:lsid:zoobank.org:pub:CFA20253-62B5-4BC0-8F30-2C054C1DB0D9. The LSID for the new name *Sphaerospora rutili* is urn:lsid:zoobank.org:act:F0840208-BE48-4913-B3AC-74C72BBBD465.
- ***Etymology*:** The species epithet “*rutili*” is derived from the type-host species name *Rutilus rutilus*.

### Description

***Spore.*** Mature spores almost spherical, with pointed apical end, measuring 8.3–9.8 × 8.8–9.3 (8.8 × 8.9) (L × T, *n* = 7) (Figs. 1l and 2h). Spores with 2 equally-sized subspherical polar capsules, measuring 3.2–4.2 × 2.7–3.5 (3.7 × 3.2) (PL × PW, *n* = 14). Polar filaments with 3–4 (*n* = 9) coils per polar capsule. Spore valves smooth, with 3–4 small humps at posterolateral end; suture line straight, pronounced, sutural ridge slightly protruding to apical spore end. Sporoplasms 2, uninucleate.

***Pseudoplasmodium.*** Mono- (Fig. 1m) and disporic (Fig. 1l) pseudoplasmodia measuring 13.7–24.0 × 7.2–17.6 (19.0 ± 2.7 × 10.4 ± 2.5) (*n* = 16), with numerous refractile granules.

### Remarks

*Sphaerospora rutili* n. sp. is morphologically similar to “Sphaerospora ousei” (*nomen nudum*) of Longshaw (2004) [44] which also possesses two uninucleated sporoplams, develops in mono- and disporic pseudoplasmodia within the renal tubules of roach. However, the ornamentation at the posterior spore end of *S. rutili* n. sp. was never observed in “S. ousei,” which has completely smooth shell valves (Table 2) [44]. Moreover, “S. ousei” has slightly elongated spores contrasting the spores of *S. rutili* n. sp. which are thicker (Table 2). Another morphologically similar species, *Sphaerospora poljanskii* Kulemina, 1969 described from the same host, differs from the present species by larger spore dimensions, a split at the apical spore end, the shape of polar capsules and by the presence of two triangular posterolateral projections (Table 2) [48]. Another roach parasite, *Sphaerospora minima* Kaschkovsky, 1974 has smaller spores and polar capsule dimensions and spine-like ornamentation arranged in three lines at the posterior spore end, contrasting *S. rutili* n. sp. spores (Table 2) [49]. A *Sphaerospora* sp. with ornamentation at the spore end and with similar spore dimensions (deduced from the figure scale-bar) was reported from the renal tubules of roach in South Bohemia, Czech Republic [50]. This is likely the same species as in present study; however, further details on spore morphology and development are missing for species comparison. Lom et al. [41] reported two undescribed *Sphaerospora* spp. from *R. rutilus* from localities in the Czech Republic. *Sphaerospora* sp. 1 has nearly identical spore and polar capsule measurements and number of polar filament coils as *S. rutili* n. sp. though their L/T ratios are distinct (Table 2). Further details about the spore surface and development are missing for species comparison. *Sphaerospora* sp. 2 is similar to the present species due to smooth spore surface, identical L/T ratio and ornamentation at the posterior end of the spore but differs by smaller spore size and higher number of polar filament coils (Table 2). Another species from roach, *Sphaerospora* sp. ex *R. rutilus* (present study) partially sequenced from Czech Republic differs by 3% (over 917 bp covering V7 and V8 regions) from *18S* rDNA sequences of *S. rutili* n. sp. Lack of morphological details impedes species comparison. Several other reports of *Sphaerospora* spp. from the blood and the kidney of roach exist but without further morphological and molecular data [42, 43, 46]. *Sphaerospora carassii* Kudo, 1919 has also been described from roach but from different organs (gills, gall-bladder and intestine) and with different spore dimensions (Table 2) [40].

### *Sphaerospora squalii* n. sp.

- ***Type-host*:** *Squalius cephalus* (L.) (Cypriniformes: Leuciscidae), European chub.
- ***Type-locality*:** River Dyje (48.6922N, 16.9184E), Czech Republic.
- ***Type-material*:** Hapantotypes: series of phototypes, deposited together with a representative DNA sample in the Protistological Collection of the Institute of Parasitology, Biology Centre, Czech Academy of Sciences, České Budějovice (accession number IPCAS Prot 52).
- ***Site in host*:** Lumen of renal tubules.
- ***Prevalence*:** 25% (1/4).
  ***Representative DNA sequence*:** A partial *18S* rDNA sequence of 3173 bp was deposited in the GenBank database under the accession number KY851780.
- ***ZooBank registration*:** To comply with the regulations set out in article 8.5 of the amended 2012 version of the *International Code of Zoological Nomenclature* (ICZN) [38], details of the new species have been submitted to ZooBank. The Life Science Identifier (LSID) of the article is urn:lsid:zoobank.org:pub:CFA20253-62B5-4BC0-8F30-2C054C1DB0D9. The LSID for the new name *Sphaerospora squalii* is urn:lsid:zoobank.org:act:6449D81B-1B2E-4B94-9293-816FDD818364.
  ***Etymology*:** The species epithet “*squalii*” is derived from the type-host species name *Squalius cephalus*.

### Description

***Spore.*** Spores almost spherical, measuring 6.1–7.1 × 6.2–7.0 (6.5 × 6.5) (L × T, *n* = 8) (Figs. 1n and 2i). Each spore with 2 equally-sized subspherical polar capsules, measuring 3.0–3.4 × 2.7–3.0 (3.2 × 2.8) (PL × PW, *n* = 16). Polar filaments with 3–4 coils (*n* = 16). Spore surface smooth, posterior end with subtle posterolateral protuberances; sutural line straight, prominent, slightly protruding at posterior end. Sporoplasms 2, uninucleate.

***Pseudoplasmodium.***Pseudoplasmodia mostly disporic and rarely monosporic, elongated, measuring 8.2–15.1 × 7.2–9.8) (10.1 × 8.3) (*n* = 13), with numerous refractile granules.

### Remarks

This is the first record of sphaerosporid spores described from *S. cephalus*. Only pseudoplasmodia were reported but further details on spore morphology and molecular data are unavailable for comparison [41]. In the present study, we found a morphologically and morphometrically similar *Sphaerospora* sp. in true minnows (Leuciscinae), i.e. *S. diversa* n. sp*.* (Table 2); however the *18S* rDNA sequences differ by 15% (see below and Additional file 7: Table S6), revealing them as two distinct species.

### Pathology

None of the screened fish showed macroscopic or microscopic pathological changes in fresh smears. Infection levels with spore-forming stages were mild and only a limited number of parasites were visible in the tubular lumen.

### *18S* rDNA sequence data

In total, 17 new *18S* rDNA sequences from 26 *Sphaerospora* (*s.s.*)-positive fish kidneys were obtained in present study (Table 1; Additional file 1: Table S1 and Additional file 6: Table S5). *18S* rDNA sequences obtained for *S. diversa* n. sp. (3 sequences from 3 host species) were complete and nine sequences were almost complete (including regions V1-V8), while another seven represent partial *18S* rDNA sequences (details in Additional file 6: Table S5). The comparison of almost complete *18S* rDNA sequences revealed that nine sphaerosporid species sampled from cypriniform hosts exceed > 3000 bp in their length while those of non-cypriniform hosts were < 3000 bp. The longest *18S* rDNA sequences were obtained for *S. gutta* n. sp. (3306 bp) and *Sphaerospora* sp. ex *C. idella* (3306 bp) (Table 1, Additional file 6: Table S5). Interspecific sequence divergence based on a trimmed alignment dataset (Additional file 7: Table S6) was overall varying to a great extent ranging from 1.87% (between *S. rutili* n. sp. and *S. dentata* n. sp.) to 59% (between *S. fugu* and *S. abrami* n. sp.), intraspecific divergence for *S. diversa* n. sp. was 0.29–0.89% (Additional file 8: Table S7). Interestingly, the interspecific divergence of sphaerosporids obtained from the same fish host was 11% (*A. brama*, covering V3-V8), 3% (*R. rutilus*, covering V7-V8; data not shown) and 15% (*S. cephalus*, covering V1-V8), respectively. *S. dentata* n. sp. and *S. gutta* n. sp. have 7% (*S. erythrophthalmus*, covering V1-V8) sequence divergence, whereas both differ 36–37% from *Sphaerospora* sp. ex *S. erythrophthalmus* (covering V5-V8) (Additional file 7: Table S6). The GC-content of the new sphaerosporid *18S* rDNA sequences varied between 48–60% over the whole *18S* rDNA and 39–73% in the variable regions (Additional file 6: Table S5). *Sphaerospora diminuta* reached the highest overall GC content (60%). *Sphaerospora elopi* n. sp. as the only new member of the “primary marine” sphaerosporid clade had a 51% overall and 49% variable region (V8) GC content. All new sphaerosporid sequences possess long insertions in the variable regions, especially in V2, V4 and V7, a characteristic feature of true sphaerosporids (Additional file 6: Table S5).

Having attempted various primer combinations, we found that the following sets most successfully amplified sphaerosporid *18S* rDNA sequences: (i) general *18S* rDNA primer combination of Erib1 + Erib10 followed by a second round PCR with a new primer combination for freshwater *Sphaerospora* spp. SphFWSSU1243F + SphFWSSU3418R (present study); (ii) a *Sphaerospora*-specific general primer combination of PsSSU1850F + Erib10 followed by a second round PCR with PsSSU2110F + Erib10 [5]; and (iii) general *18S* rDNA primer combination of Erib1 + Erib10 followed by second round PCR with MyxGP2F + Act1R [14, 24] (details in Additional file 2: Table S2). A combination of expanded primer extension time and highly efficient TITANIUM Taq polymerase considerably improved the outcome of PCRs.

### Phylogenetic relationships within the *Sphaerospora* (*s.s.*) clade

**Fig. 3:**
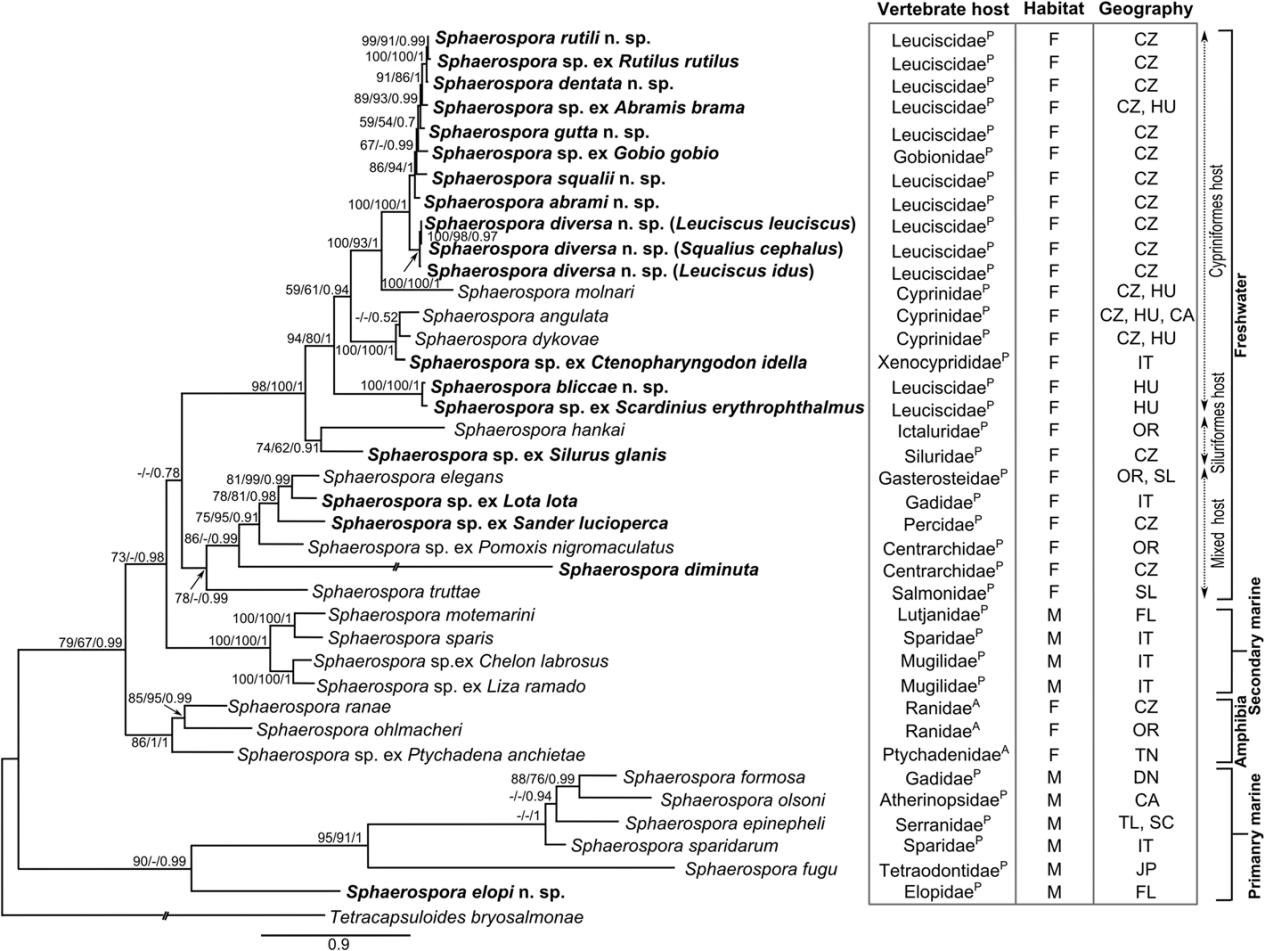
*18S* rDNA-based maximum likelihood (GTR + Γ model) tree of the *Sphaerospora* (*sensu stricto*) clade. *Tetracapsuloides bryosalmonae* was used as the outgroup. Newly sequenced taxa are in bold. Maximum likelihood/maximum parsimony/Bayesian inference nodal supports are shown at every node. Dashes indicate < 50 nodal support values or a node missing in the maximum parsimony and Bayesian inference analyses. The original length of long branches was shortened to 50% of their original length (indicated as -//-). Superscript letters at the end of the vertebrate host families indicate the host types (P, piscine; and A, amphibian). *Abbreviations*: F, freshwater; M, marine; CA, California, USA; CZ, Czech Republic; DN, Denmark; FL, Florida, USA; HU, Hungary; IT, Italy; JP, Japan; OR, Oregon, USA; SC, South China Sea; SL, Scotland; TL, Thailand; TN, Tanzania

**Fig. 4:**
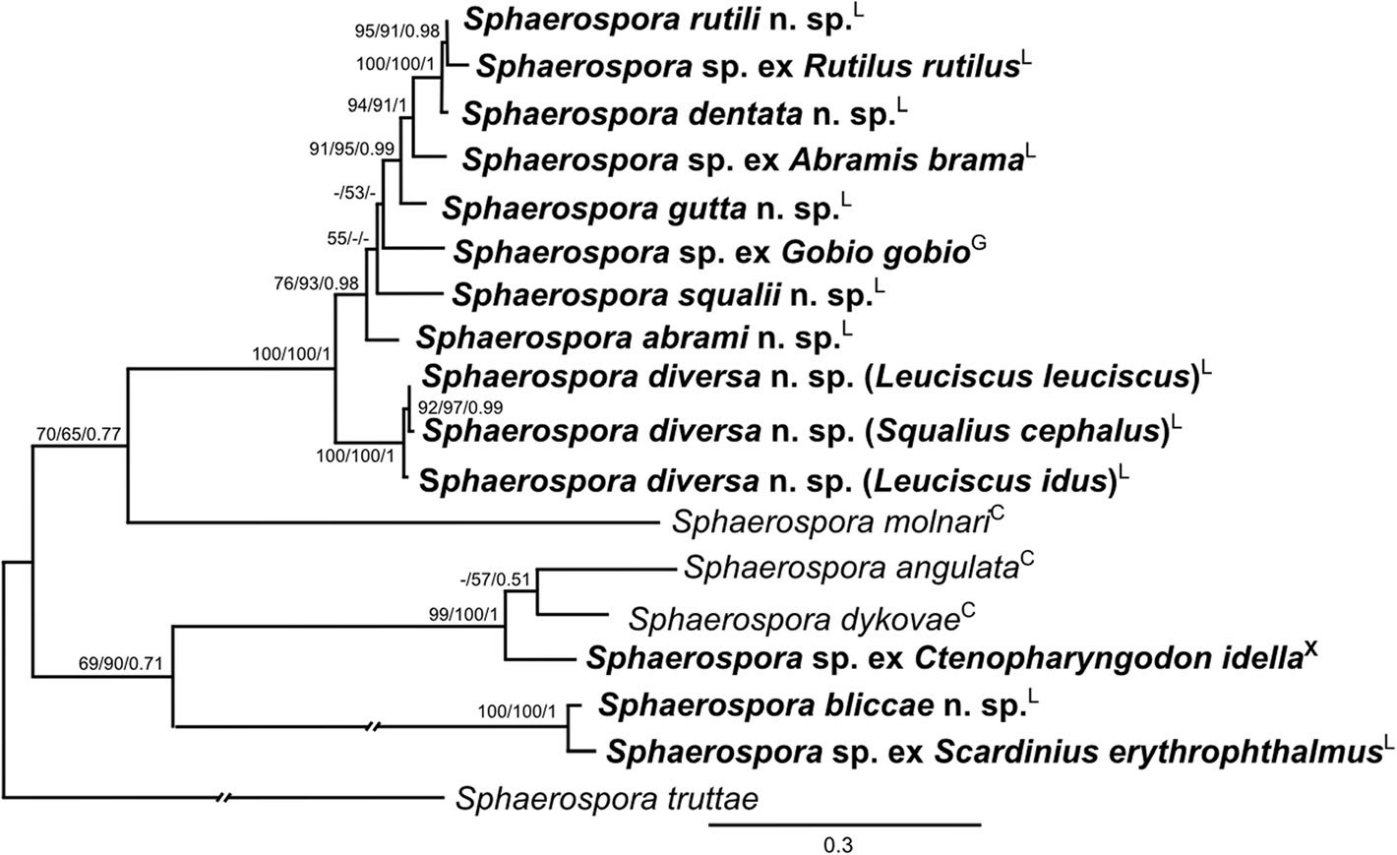
*18S* rDNA-based maximum likelihood (GTR + Γ model) tree of *Sphaerospora* spp. from fish from the order Cypriniformes, with *Sphaerospora truttae* used as the outgroup. Newly sequenced taxa are in bold. Maximum likelihood/maximum parsimony/Bayesian inference support values are shown at every node. Dashes indicate < 50 nodal support values or a node missing in the maximum parsimony and Bayesian inference analyses. The original length of long branches was shortened to 50% of their original length (indicated as -//-). Superscript letters at the end of the species names indicate their attribution to the particular family (C, Cyprinidae; G, Gobionidae; L, Leuciscidae; X, Xenocyprididae)

The phylogenetic tree of *18S* rDNA sequences including all newly sequenced taxa (Fig. 3) shows that all new sequences cluster within the *Sphaerospora* (*s.s.*) clade, allowing us to consider them “true sphaerosporids”. The new sequences cluster into two distinct clades: (i) a basal “primary marine” clade of sphaerosporids from marine teleosts (i.e. Lineage A in [5]); and (ii) all other sphaerosporids (i.e. Lineage B in [5]). The latter, larger clade is subdivided into 3 distinct subclades (Fig. 3): (i) a clade of sphaerosporids from amphibians; (ii) the “secondary marine” clade of sphaerosporids with spores containing 4–12 sporoplasms (*vs* otherwise commonly 2) from marine habitats; and (iii) a “freshwater clade” of sphaerosporids from freshwater fishes, which includes the type-species *S. elegans.* The freshwater clade is further divided into three subclades including: (i) sphaerosporids from cypriniform hosts; (ii) species from siluriform hosts; and (iii) a subclade of species from mixed fish host families. *Sphaerospora molnari,* the only histozoic parasite of the freshwater clade for which *18S* rDNA sequences are available, creates a distinct sublineage. *Sphaerospora diminuta* produces a long branch within the mixed host freshwater subclade. Geography did not reflect phylogenetic clustering of sphaerosporids; however, host habitat (freshwater *vs* marine) and host group (at the ordinal level) showed a clear pattern in certain clades. Sphaerosporids from the same host order clustered together in the same clade or in sister clades (e.g. Cypriniformes, Centrarchiformes, Mugiliformes, Siluriformes and Anura) (Fig. 3). However, this trend was not observed at host family level as, for example, sphaerosporids from Gobionidae and Xenocyprididae grouped inside species of Leuciscidae and Cyprinidae, respectively (Fig. 4). Moreover, sphaerosporids from Leuciscidae and Cyprinidae clustered in more than one clade within the phylogenetic tree.

### Cophylogeny analyses

The phylogenetic analysis of vertebrate mitochondrial sequence data revealed a tree topology that is in accordance with recent phylogenomic studies [51, 52], apart from the position of *Takifugu rubripes* (Eupercaria: Tetraodontiformes: Tetraodontidae) which clustered outside Eupercaria and basal of Percomorphaceae. However, we did not exclude this species from tree reconciliation analysis. In the sphaerosporid phylogenetic tree used for cophylogeny, species clustering was unaltered after excluding six species (see “Cophylogenetic analyses” in Methods section; Additional file 9: Table S8).

**Fig. 5:**
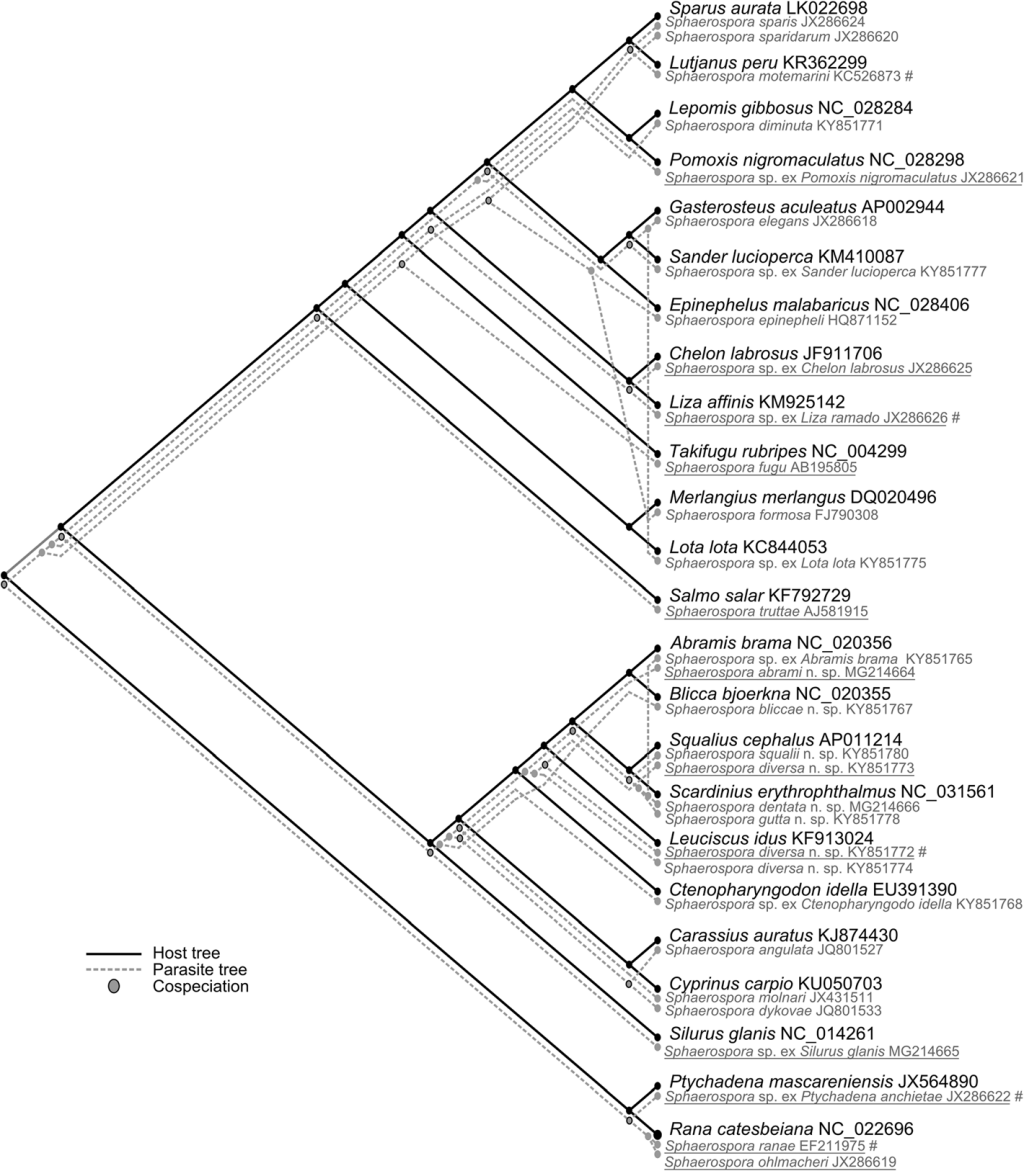
Cophylogeny reconstruction of *Sphaerospora* spp. (*18S* rDNA sequences) and their vertebrate hosts (full mitogenome sequences), using CoRe-PA. Vertebrate maximum likelihood (ML) tree shown in black and parasite ML tree as grey dashed lines. # indicates that sequence data of a closely related vertebrate host were used for the analysis, as complete mitogenome data were unavailable for this specific host. Underlined names indicate host-parasite cophylogeny detected by ParaFit

The tree topology-based analysis performed in CoRe-PA detected significant congruence between the phylogenetic trees of sphaerosporids and their vertebrate hosts (Fig. 5), with 18 cospeciation events (estimated cost for cospeciation = 0.105) calculated from a dataset of 24 hosts and 31 parasites. Quality of the reconstruction was 1.8830165 × 10^-11^ with a total cost of 7.578. CoRe-PA estimated 35 sorting (cost 0.054) and three host switching (cost 0.631) events: (i) from *S. erythrophthalmus* to *A. brama*; (ii) from *Gasterosteus aculeatus* L. to *L. lota*; and (iii) from a common perciform ancestor to *Merlangius merlangus* (L.), where all parasites established and diversified successfully. In cypriniforms, sphaerosporid diversity is presently the highest, based on the sampling performed in this study. In half of investigated cypriniform hosts, two independent sphaerosporid lineages are present. Furthermore, the analysis showed that the oldest cypriniforms already had three independent parasite lineages, indicating an extremely successful radiation of sphaerosporids in this host group. Global fit analysis detected 19 (F1.stat) or 24 (F2.stat) statistically significant coevolving host-parasite pairs, depending on the statistics used (calculating ligand-receptor relation importance in F1.stat or using a non-permutated matrix in F2.stat), and resulting in a global fit of 0.2281377 with highly significant *P*-value of 0.001 over 999 permutations (Additional file 10: Table S9).

## Discussion

### *Sphaerospora* (*s.s.*) biodiversity, phylogeny and host specificity

Due to the difficulty of amplifying the strongly divergent sequences and extremely long, species-specific insertions by PCR, a condition that is further complicated by myxozoan co-infections in kidneys, *Sphaerospora* (*s.s.*) *18S* rDNA sequence data has long been scarce [5]. Based on the development of new primers ([5], present study), the *Sphaerospora* (*s.s.*) clade was enlarged from 19 [5, 7, 9, 53] to 36 species. Based on *18S* rDNA sequence divergence criteria proposed for other myxozoans [54–56], < 1% divergence was considered conspecific for *S. diversa* n. sp. (3 sequences; this study) and > 1% was considered interspecific variation [56]. However, species-specific long insertions in *18S* rDNA cause extremely high sequence divergence (1.87–59.00%) in sphaerosporids, thereby greatly facilitating the differentiation, even of closely related species. Phylogenetic analyses of the enriched dataset showed clustering of the newly obtained sequences in previously established clades, and their GC content matched the previously recognized difference for the two main sphaerosporid clades [5]. However, some additional key findings were revealed in this study. *Sphaerospora elopi* n. sp. from an evolutionary older teleost, *E. saurus* (Elopiformes), represents presently the most basal species of “primary marine” sphaerosporids. The “anadromous host” clade of Bartošová et al. [5] was enriched by *Sphaerospora* spp. from freshwater fishes *L. lota*, *S. lucioperca* and *L. gibbosus*. *Sphaerospora truttae* is the only species with anadromous hosts (*Salmo salar* L. and *Salmo trutta* L.) in this clade but infects its hosts only in freshwater [57]. Moreover, since *S. elegans 18S* rDNA was sequenced from *Gasterosteus aculeatus* from an isolated freshwater site (A. Holzer, pers. comm.) and *Pomoxis nigromaculatus* (Lesueur, 1829) is a freshwater species, this clade can be considered as a “true” freshwater clade, justifying the changed attribute “mixed host clade”. Important biodiversity and data enrichment for sphaerosporids from cypriniform hosts (12 new species) allows the interpretation of the clustering of a large number of sphaerosporids from closely related hosts and statements on host specificity. The long branch created by *S. diminuta* probably represents a novel sublineage rather than a phylogenetic artefact, as variable regions (specifically V4 and V5) and GC content are distinct from the rest of the other sphaerosporids (Additional file 6: Table S5). However, *Sphaerospora* sp. from *P. nigromaculatus* clusters sister to *S. diminuta*, from another centrarchiform host, *L. gibbosus*. Further taxon sampling from this fish family could resolve the long branch position of *S. diminuta* in the future.

Central European cypriniforms were suggested as a *Sphaerospora* biodiversity hostspot [41, 42] long before DNA sequencing became accessible. Our molecular data confirm this observation, further suggesting that even morphologically indistinguishable species from different hosts [24] can represent different species. DNA sequencing demonstrates rather strict host specificity as a general rule in sphaerosporids ([5, 24], present study). Hence, reports of single species in multiple unrelated hosts [40, 58–61] require confirmation by DNA sequencing. Strict host specificity has few exceptions, but only involves closely related hosts: *Sphaerospora epinepheli* Supamattaya, Fischer-Scherl, Hoffmann & Boonyaratpalin, 1991 in *Epinephelus malabaricus* (Block & Schneider, 1801) and *Epinephelus coioides* (Hamilton, 1822) [53, 62], *Sphaerospora angulata* Fujita, 1912 in *Carassius auratus* (L.) and *Carassius gibelio* (Bloch, 1782) [24], *S. truttae* in *S. salar* and *S. trutta* [14, 63], and *S. diversa* n. sp. in three closely related [64] leuciscinids (present study). Interestingly, identical *18S* rDNA sequences of *S. diversa* were obtained from two *L. leuciscus* specimens of different geographical origin (Table 1) but intraspecific sequences divergence from all three hosts ranged between 0.29–0.89% (Additional file 8: Table S7). These differences may well represent another diversification step of sphaerosporids within leuciscinid hosts, especially because the sphaerosporid sequences from *L. leuciscus* and *S. cephalus* were obtained from the same sampling site (Table 1), hence potentially indicating first stages of host-mediated diversification, as observed in *Myxobolus pseudodispar* Gorbunova, 1936 [65]. Cypriniforms are hosts of multiple sphaerosporids (Table 1), potentially indicating hyperdiversification of sphaerosporids in cypriniforms, which is also highly pronounced in myxobolids [16].

### Biological characteristics mirroring phylogenetic relationships of *Sphaerospora* (*s.s.*)

Tissue tropism-dependent phylogenetic clustering is the most common phenomenon in myxozoans, defining a large number of subclades (reviewed by [2]). However, sphaerosporids are predominantly urinary tract-infecting parasites, with only two histozoic members sequenced to date, *S. fugu* from the intestinal epithelium [66] and *S. molnari* from the gill epithelium [67]. All members of the most basal myxozoan clade, the Malacosporea, are coelozoic in the renal tubules (reviewed by [56]), and sphaerosporids share this location as their preferred intrapiscine site. This indicates that renal tubules are an ancestral localization [5, 68]. In sphaerosporids, a switch to a histozoic development happened at least twice independently, in *S. fugu* and in *S. molnari* [4, 9]. Various sphaerosporids have been reported from unusual sites such as the eye [40], gall-bladder [69–73], gills [40, 67, 74, 75], intestine [40, 51, 69, 76–78], muscle [58], oral cavity [58], ovary [37, 79, 80] and skin [75], without molecular characterisation. Although the present study was focused on the urinary system, the main infection site of sphaerosporids in fish, the abovementioned organs should be analysed in further *Sphaerospora* molecular research. Sequence data from these species could determine how many times the histozoic type of development evolved in sphaerosporids and if tissue tropism-related phylogenetic clustering exists. The lack of tissue-tropism diversity in sphaerosporids led us to investigate other patterns such as geography, which was not found to mirror the phylogenetic tree of this group (Fig. 3) though important for the clustering of other myxozoans, especially at the species level [54, 81–83]. Host habitat and host order clearly reflect sphaerosporid clustering, similar to other myxozoan clades (e.g. [84–86]).

### Coevolution of species of *Sphaerospora* (*s.s.*) and their vertebrate hosts

Phylogenetic clustering of sphaerosporids according to host order led us to investigate host-parasite codivergence in this clade of myxozoans and to unravel the evolutionary history of sphaerosporids. Cophylogenetic analyses showed highly significant congruence between the phylogeny of sphaerosporids and their vertebrate hosts, by both, tree topology-based and distance-based methods. Although distance-based methods are considered less biased [87], using a smaller dataset of 19 hosts and 19 sphaerosporids [16] did not result in a significant outcome when using *16S* mtRNA data, likely because this limited host dataset showed similar distances between taxa. Holzer et al. [16] showed that full mitogenome host data improve the outcome of distance-based methods but had only limited parasite sequences available and mitogenome data was not analysed at the species level. In our mitogenome-based host phylogeny, all taxa except *Takifugu rubripes* (Temminck & Schlegel) clustered according to the most updated fish phylogeny inferred using genomic data of nearly 2000 fishes [52]. The improved taxon sampling and more informative host dataset used in the present study hence considerably improved the outcome of cophylogenetic studies. Especially interesting is the finding that cypriniforms are a “preferred” host group with multiple parasite lineages in individual hosts. This appears to support the finding that hyperdiverse host fish groups (Ostariophysi and Percomorpha) [88] show a pronounced potential for parasite diversification [16], also in sphaerosporids. A higher potential of parasite sharing between closely related hosts [89, 90] and host-driven diversification was observed in *Sphaerospora* spp. in leuciscinids in the present study. Closely related cypriniforms are among the most abundant fish groups in European freshwaters [91, 92], often sharing the same habitat. This allows diversification of relatively host-specific taxa such as *Sphaerospora* (*s.s.*) spp., hence explaining the high biodiversity of sphaerosporids in these habitats, though sampling bias cannot be excluded at present [93].

### Evolutionary history of sphaerosporids and their alternate hosts

Holzer et al. [16] suggested that sphaerosporids likely have a marine origin and may have settled in “archiannelid” (chaetopterids or sipunculids) invertebrate hosts. The present study appears to further indicate the presence of two independent entries of sphaerosporids into archiannelids: (i) at the base of the primary marine clade; and (ii) at the root of all other sphaerosporids. This suggestion is based on the observation that elopiform fishes (Teleostei) are the oldest vertebrate hosts in the primary marine clade [51, 52] while tetrapods, which originated earlier than teleosts, occupy this position in the large clade harbouring all other *Sphaerospora* spp. It is possible that the archiannelid acquired as host in the primary marine lineage was maintained as a single host until teleosts evolved in the marine realm, while the large sphaerosporid clade appears to have a similar evolutionary history as most other myxozoan clades which accommodate cartilaginous fish as their first host group [94–96], followed by lineages in tetrapods and finally mirroring the evolution of teleosts [16]. To support this idea, it would be essential to sequence sphaerosporids from evolutionary old fish lineages such as the Chondrichthyes or even the Cyclostomata. A single species, *Sphaerospora araii* Arthur & Lom, 1985 was described from a ray, *Raja rhina* Jordan & Gilbert, 1880 [78], but our newly developed primer sets may be able to uncover and sequence further species in cartilaginous fishes. We believe that sphaerosporids from cartilaginous fishes represent missing links that would be able to confirm phylogenetic congruence of sphaerosporids and their vertebrate hosts and contribute further information on their common evolutionary history.

## Conclusions

The present study aimed at elucidating the phylogeny and evolutionary history of *Sphaerospora* (*s.s.*), based on a greatly enlarged (almost doubled) dataset of difficult to amplify *18S* rDNA sequences. Larger datasets including information of new host groups and habitats provided important data, explaining parasite phylogenetic clustering. We report a very narrow host specificity for sphaerosporids. *Sphaerospora diversa* n. sp. sequenced from three closely related leuciscinid species showed low sequence divergences, presumably reflecting initial host-driven diversification while the remainder of the newly sequenced species were strictly host-specific. Cypriniforms are characterized by multiple parasite lineages, indicating successful parasite diversification within this host group. Cophylogenetic analyses revealed significant phylogenetic congruence between sphaerosporids and their vertebrate hosts. Based on cophylogenetic analysis, we suggest that parasite entry to invertebrate hosts occurred twice independently during sphaerosporid evolution. Sequencing of sphaerosporids from cartilaginous fish, or other evolutionary older vertebrate groups could substantially support this idea and further elucidate the evolutionary history of this group of fast evolving myxozoans.

## Additional files

Additional file 1: **Table S1.** List of fish hosts examined for *Sphaerospora* spp. infections with information about locality, number of fish examined, parasite prevalence (positive samples in a black box) and estimated age from the total length. (DOCX 51 kb)

Additional file 2: **Table S2.** List of PCR primer combinations and other PCR details applied to amplify *18S* rDNA sequences of different sphaerosporids. (DOCX 50 kb)

Additional file 3: **Table S3.** Newly designed primers used in this study. (DOCX 28 kb)

Additional file 4: **Table S4.** PCR cycling parameters used for sphaerosporid amplification. (DOCX 28 kb)

Additional file 5. *18S* rDNA dataset (.fasta format) of 4813 bp long *Sphaerospora* (*s.s.*) sequences. (FASTA 190 kb)

Additional file 6: **Table S5.** The lengths of variable regions (base pairs) and GC-content (percentage; in parenthesis) of newly obtained *18S* rDNA *Sphaerospora* sequences (bold numbers indicate the highest values). (DOCX 32 kb)

Additional file 7: **Table S6.** *18S* rDNA sequence dissimilarities of *Sphaerospora* (*s.s.*) species. Almost complete sequences are compared only. (XLSX 15 kb)

Additional file 8: **Table S7.** *18S* rDNA sequence dissimilarities (based on complete sequence dataset of 3112 bp) of *Sphaerospora diversa* n. sp. from three host species. (XLSX 9 kb)

Additional file 9: **Table S8**. List of members of *Sphaerospora* (*s.s.*) clade (*18S* rDNA), their vertebrate hosts (complete mitogenome) and GenBank accession numbers (*18S* rDNA for sphaerosporids and complete mitogenome for vertebrate host) used for cophylogeny studies (CoRe-Pa and Parafit analysis). (DOCX 33 kb)

Additional file 10: **Table S9.** ParaFit analysis (performed in APE package v3.4 in R v3.2.4) result for sphaerosporids and their vertebrate hosts. Statistically significant results (< 0.050) are indicated in bold. Test statistics were calculated (i) between the of sums of squares of values in the main diagonal of the combined host-parasite matrix and a matrix with unspecific interaction to estimate the ligand-receptor relation which is more restricted and preferred value (F1.stat), and (ii) the difference calculated by the trace of non-permutated matrix (F2.stat). (DOCX 34 kb)

## Abbreviations

BI: Bayesian inference
LSID: Life Science Identifier
ML: Maximum likelihood analysis
MP: Maximum parsim">Polar capsule length
PW: Polar capsule width
L: Spore length
T: Spore thickness
L/T ratio: Spore length to thickness ratio

## References

[1] Zhang ZQ (2011). Animal biodiversity: An introduction to higher-level classification and taxonomic richness. Zootaxa..

[2] Fiala I, Bartošová-Sojková P, Whipps CM, Okamura B, Gruhl A, Bartholomew JL (2015). Classification and phylogenetics of Myxozoa. Myxozoan evolution, ecology and development.

[3] Lom J, Dyková I (2006). Myxozoan genera: definition and notes on taxonomy, life-cycle terminology and pathogenic species. Folia Parasitol..

[4] Jirků M, Fiala I, Modrý D (2007). Tracing the genus *Sphaerospora*: rediscovery, redescription and phylogeny of the *Sphaerospora ranae* (Morelle, 1929) n. comb. (Myxosporea, Sphaerosporidae), with emendation of the genus *Sphaerospora*. Parasitology..

[5] Bartošová P, Fiala I, Jirků M, Cinková M, Caffara M, Fioravanti ML (2013). *Sphaerospora sensu stricto*: Taxonomy, diversity and evolution of a unique lineage of myxosporeans (Myxozoa). Mol Phylogenet Evol..

[6] Holzer AS, Pecková H, Patra S, Brennan NP, Yanes-Roca C, Main KL (2013). Severe glomerular disease in juvenile grey snapper *Lutjanus griseus* L. in the Gulf of Mexico caused by the myxozoan *Sphaerospora motemarini* n. sp. Int J Parasitol Parasites Wildl..

[7] Sanders JL, Jaramillo AG, Ashford JE, Feist SW, Lafferty KD, Kent ML (2015). Two myxozoans from the urinary tract of topsmelt, *Atherinops affinis*. J Parasitol.

[8] Holzer AS, Wootten R, Sommerville C (2007). The secondary structure of the unusually long *18S* ribosomal RNA of the myxozoan *Sphaerospora truttae* and structural evolutionary trends in the Myxozoa. Int J Parasitol..

[9] Eszterbauer E, Sipos D, Forró B, Bartošová P, Holzer A (2013). Molecular characterization of *Sphaerospora molnari* (Myxozoa), the agent of gill sphaerosporosis in common carp *Cyprinus carpio*. Dis Aquat Organ..

[10] Jirků M, Bartošová-Sojková P (2014). Ultrastructure and localisation of late-sporogonic developmental stages of *Sphaerospora ranae* (Myxosporea: Sphaerosporidae). Folia Parasitol..

[11] Özer A, Wootten R. The life cycle of *Sphaerospora truttae* (Myxozoa: Myxosporea) and some features of the biology of both the actinosporean and myxosporean stages. Dis Aquat Organ. 2000;40:33–9. 10.3354/dao040033 10785861

[12] Molnár K, El-Mansy A, Székely C, Baska F. Experimental identification of the actinosporean stage of *Sphaerospora renicola* Dyková & Lom, 1982 (Myxosporea: Sphaerosporidae) in oligochaete alternate hosts. J Fish Dis. 1999;22:143–53.

[13] Holzer AS, Sommerville C, Wootten R (2004). Molecular relationships and phylogeny in a community of myxosporeans and actinosporeans based on their *18S* rDNA sequences. Int J Parasitol..

[14] Eszterbauer E, Marton S, Rácz OZ, Letenyei M, Molnár K (2006). Morphological and genetic differences among actinosporean stages of fish-parasitic myxosporeans (Myxozoa): difficulties of species identification. Sys Parasitol..

[15] Desser SS, Lom J, Dyková I (1986). Developmental stages of *Sphaerospora ohlmacheri* (Whinery, 1893) n. comb. (Myxozoa:Myxosporea) in the renal tubules of bullfrog tadpoles, *Rana catesbeiana*, from Lake of Two Rivers, Algonquin Park, Ontario. Can J Zool..

[16] Holzer AS, Bartošová-Sojková P, Born-Torrijos A, Lövy A, Hartigan A, Fiala I. The joint evolution of the Myxozoa and their alternate hosts: a cnidarian recipe for success and vast biodiversity. Mol Ecol. 2018;27:1651–66. 10.1111/mec.14558 29575260

[17] Jiménez-Guri E, Philippe H, Okamura B, Holland PWH (2007). *Buddenbrockia* is a cnidarian worm. Science..

[18] Evans NM, Holder MT, Barbeitos MS, Okamura B, Cartwright P (2010). The phylogenetic position of Myxozoa: exploring conflicting signals in phylogenomic and ribosomal data sets. Mol Biol Evol..

[19] Chang ES, Neuhof M, Rubinstein ND, Diamant A, Philippe H, Huchon D (2015). Genomic insights into the evolutionary origin of Myxozoa within Cnidaria. Proc Natl Acad Sci USA..

[20] Takeuchi F, Sekizuka T, Ogasawara Y, Yokoyama H, Kamikawa R, Inagaki Y (2015). The mitochondrial genomes of a myxozoan genus *Kudoa* are extremely divergent in Metazoa. PLoS One..

[21] Lom J, Arthur JR (1989). A guideline for the preparation of species descriptions in Myxosporea. J Fish Dis..

[22] Sitja-Bobadilla A, Alvarez-Pellitero P (1994). Revised classification and key species of the genus *Sphaerospora* Davies, 1917 (Protozoa: Myxosporea). Res Rev Parasitol..

[23] Asahida T, Kobayashi T, Saitoh K, Nakayama I (1996). Tissue preservation and total DNA extraction from fish stored at ambient temperature using buffers containing high concentration of Urea. Fish Sci..

[24] Holzer AS, Bartošová P, Pecková H, Tyml T, Atkinson S, Bartholomew J, et al. ‘Who’s who’ in renal sphaerosporids (Bivalvulida: Myxozoa) from common carp, Prussian carp and goldfish - molecular identification of cryptic species, blood stages and new members of *Sphaerospora sensu stricto*. Parasitology. 2013;140:46–60. 10.1017/S0031182012001175 22917178

[25] Katoh K, Misawa K, Kuma K, Miyata T (2002). MAFFT: a novel method for rapid multiple sequence alignment based on fast Fourier transform. Nucleic Acids Res..

[26] Kearse M, Moir R, Wilson A, Stones-Havas S, Cheung M, Sturrock S (2012). Geneious Basic: an integrated and extendable desktop software platform for the organization and analysis of sequence data. Bioinformatics..

[27] Stamatakis A (2006). RAxML-VI-HPC: Maximum likelihood-based phylogenetic analyses with thousands of taxa and mixed models. Bioinformatics..

[28] Posada D (2008). JModelTest: phylogenetic model averaging. Mol BIol Evol..

[29] Swofford DL (2002). PAUP*. Phylogenetic Analysis Using Parsimony (*and other methods). Version 4.

[30] Ronquist F, Huelsenbeck JP (2003). MrBayes 3: Bayesian phylogenetic inference under mixed models. Bioinformatics..

[31] Rambaut A, Suchard MA, Xie D, Drummond AJ (2014). Tracer v1.6.

[32] Merkle D, Middendorf M, Wieseke N (2010). A parameter-adaptive dynamic programming approach for inferring cophylogenies. BMC Bioinformatics..

[33] Legendre P, Desdevises Y, Bazin E (2002). A statistical test for host-parasite coevolution. Syst Biol..

[34] Paradis E, Claude J, Strimmer K (2004). APE: analyses of phylogenetics and evolution in R language. Bioinformatics..

[35] Li LX, Desser SS (1985). The protozoan parasites of fish from two lakes in Algonquin Park. Ontario. Can J Zool..

[36] Lom J, Desser SS, Dyková I (1989). Some little-known and new protozoan parasites of fish from Lake Sasajewun, Algonquin Park, Ontario. Can J Zool.

[37] Xiao C, Desser SS. *Sphaerospora ovophila* n. sp. and *Myxobolus algonquinensis* n. sp. (Myxozoa, Myxosporea), ovarian parasites of fish from Algonquin Park, Ontario, Canada. J Eukaryot Microbiol. 1997;44:157–61.

[38] ICZN (2012). International Commission on Zoological Nomenclature: Amendment of articles 8, 9, 10, 21 and 78 of the International Code of Zoological Nomenclature to expand and refine methods of publication. Bull Zool Nomencl..

[39] El-Matbouli M, Hoffmann R, Kern R (1995). *Sphaerospora bramae* sp. nov. (Myxosporea: Sphaerosporidae) in the kidney of common bream (*Abramis brama*). Bull Eur Ass Fish Pathol..

[40] Kudo R (1919). Studies on Myxosporidia. A synopsis of genera and species of Myxosporidia. Ill Biol Monogr..

[41] Lom J, Pavlásková M, Dyková I (1985). Notes on kidney-infecting species of the genus *Sphaerospora* Thélohan (Myxosporea), including a new species *S. gobionis* sp. nov., and on myxosporean life cycle stages in the blood of some freshwater fish. J Fish Dis..

[42] Baska F, Molnár K (1988). Blood stages of *Sphaerospora* spp. (Myxosporea) in cyprinid fishes. Dis Aquat Organ..

[43] El-Matbouli M, Hoffmann RW (1992). *Sphaerospora scardinii* n. sp. (Myxosporea, Sphaerosporidae) observed in the kidney of rudd *Scardinius erythrophthalmus*. Dis Aquat Organ..

[44] Longshaw M (2004). Studies of myxozoan parasites of freshwater fish and invertebrate hosts. PhD Thesis.

[45] Zaika VE (1961). The question of endemicity among the parasites of the fishes in Lake Baikal. Dokl Akad Nauk SSSR..

[46] Molnár K (1980). Renal sphaerosporosis in the common carp *Cyprinus carpio* L. J Fish Dis..

[47] Hanajavanit C, Bermingham M, Mulcahy MF (2008). Epidemiology of squamous cell carcinomas in rudd *Scardinius erythrophthalmus* from SE Ireland. Dis Aquat Organ..

[48] Kulemina IV (1969). New species of endoparasitic protozoa from the fry of the Lake Seliger. Zool Zh..

[49] Kashkovsky VV, Razmashkin DA, Skripchenko GE (1974). Diseases and parasites of fishes in Siberian and Ural fish farms.

[50] Kepr T. Parasitic Protozoa of cyprinid fishes: Protozoa of the roach *Rutilus rutilus* (Linnaeus, 1758) in Czechoslovakia. Folia Parasitol. 1991;38:11–21. 1916526

[51] Near TJ, Eytan RI, Dornburg A, Kuhn KL, Moore JA, Davis MP (2012). Resolution of ray-finned fish phylogeny and timing of diversification. Proc Natl Acad Sci USA..

[52] Betancur-R R, Wiley EO, Arratia G, Acero A, Bailly N, Miya M (2017). Phylogenetic classification of bony fishes. BMC Evol Biol..

[53] U-Taynapun K, Chirapongsatonkul N, Maneesaay P, Itami T, Tantikitti C (2012). A new host record of *Sphaerospora epinepheli* (Myxosporea: Bivalvulida) occurring on orange-spotted grouper *Epinephelus coioides* from Thailand: epidemiology, histopathology and phylogenetic position. Vet Parasitol..

[54] Whipps CM, Kent ML (2006). Phylogeography of the cosmopolitan marine parasite *Kudoa thyrsites* (Myxozoa: Myxosporea). J Eukaryot Microbiol..

[55] Bartošová P, Fiala I (2011). Molecular evidence for the existence of cryptic species assemblages of several myxosporeans (Myxozoa). Parasitol Res..

[56] Patra S, Hartigan A, Morris DJ, Kodádková A, Holzer AS (2017). Description and experimental transmission of *Tetracapsuloides vermiformis* n. sp. (Cnidaria: Myxozoa) and guidelines for describing malacosporean species including reinstatement of *Buddenbrockia bryozoides* n. comb. (syn. *Tetracapsula bryozoides*). Parasitology..

[57] McGeorge JM, Sommerville C, Wootten R (1996). Epizootiology of *Sphaerospora truttae* (Myxozoa: Myxosporea) infections of Atlantic salmon *Salmo salar* at freshwater smolt producing hatcheries in Scotland. Dis Aquat Organ..

[58] Fantham HB, Porter A, Richardson LR. Some myxosporidia found in certain freshwater fishes in Quebec Province, Canada. Parasitology. 1939;31:1–77.

[59] Shul’man SS (1966). Myxosporidia of the USSR. Moscow-Lenningrad: Nauka Publishers. Translated version published for the United States Department of the Interior and National Science Foundation.

[60] Molnár K (1991). *Sphaerospora danubialis* sp. n. (Myxosporea: Sphaerosporidae) from the kidney of freshwater percid fishes. Parasitol Hung..

[61] Fomena A, Bouix G (1994). New Myxosporidea species (Myxozoa) from freshwater teleosts in southern Cameroon (Central Africa). J Afr Zool..

[62] Supamattaya K, Fischerscherl T, Hoffmann RW, Boonyaratpalin S (1991). *Sphaerospora epinepheli* n. sp. (Myxosporea: Sphaerosporidae) observed in grouper (*Epinephelus malabaricus*). J Protozool..

[63] Holzer AS, Sommerville C, Wootten R (2006). Molecular studies on the seasonal occurrence and development of five myxozoans in farmed *Salmo trutta* L. Parasitology..

[64] Stout CC, Tan M, Lemmon AR, Lemmon EM, Armbruster JW (2016). Resolving Cypriniformes relationships using an anchored enrichment approach. BMC Evol Biol..

[65] Forró B, Eszterbauer E (2016). Correlation between host specificity and genetic diversity for the muscle-dwelling fish parasite *Myxobolus pseudodispar*: examples of myxozoan host-shift?. Folia Parasitol..

[66] Tun T, Yokoyama H, Ogawa K, Wakabayashi H (2000). Myxosporeans and their hyperparasitic microsporeans in the intestine of emaciated tiger puffer. Fish Pathol..

[67] Lom J, Dyková I, Pavlásková M, Grupcheva G (1983). *Sphaerospora molnari* sp. n. (Myxozoa, Myxosporea), an agent of gill, skin and blood sphaerosporosis of common carp in Europe. Parasitology..

[68] Fiala I, Bartošová P (2010). History of myxozoan character evolution on the basis of rDNA and EF-2 data. BMC Evol Biol..

[69] Cohn L (1902). Zur Kenntniss der Myxosporidien. Zentralbl Bakter Parasitenkd.

[70] Fantham HB, Porter A (1943). On a myxosporidian. *Sphærospora periophthalmi,* sp. n., found in African and Indian Mudskippers (Pisces), and its possible significance. Proc Zool Soc Lond..

[71] Moser M, Kent ML, Dennis D (1989). Gall-bladder Myxosporea in coral-reef fishes from Heron Island, Australia. Aust J Zool.

[72] Sarkar NK, Ghosh S (1991). Two new coelozoic Myxosporida (Myxozoa: Myxosporea) from estuarine teleost fishes (Mugilidae) of West Bengal, India. Proc Zool Soc Calcutta.

[73] Brickle P, Kalavati C, MacKenzie K (2001). Two new species of myxozoan parasites (Myxosporea, Bivalvulida) from toothfish *Dissostichus eleginoides* Smitt, 1898 (Pisces, Nototheniidae). Acta Parasitol..

[74] Su X, White RWG (1994). New myxosporeans (Myxozoa: Myxosporea) from marine fishes of Tasmania, Australia. Acta Protozool.

[75] Chen C, Ma C (1998). Myxozoa, Myxosporea. Fauna Sinica.

[76] Jacob E, Bremen HB (1953). Eine bislang unbekannte Sphaerosporose des Flussaals, hervorgerufen durch *Sphaerospora reichenowi* nova species, mit eigenartigem Sitz im Darm. Berl Munch Tierarztl Wochenschr..

[77] Chen C, Hsieh S (1984). A new genus and two new species of family Myxobolidae from freshwater fishes of China (Myxosporidia: Myxobolidae). Acta Zootaxon Sinica..

[78] Arthur JR, Lom J (1985). *Sphaerospora araii* n. sp. (Myxosporea: Sphaerosporidae) from the kidney of a longnose skate (*Raja rhina* Jordan and Gilbert) from the Pacific Ocean off Canada. Can J Zool..

[79] Schuurmans-Stekhoven JJH (1920). Über einige Myxosporidien des Stichlings. Arch Protistenk..

[80] Moshu AY. Description of *Sphaerospora luciopercae* sp. n. (Protista: Myxosporea) - parasite of the European pikeperch *Stizostedion lucioperca* (L.). Bul Akad Stiinte Republicci Moldova. Stinnte Biologice si Chimice. 1992;2:54–6.

[81] Whipps CM, Adlard RD, Bryant MS, Lester RJG, Findlay V, Kent ML (2003). First report of three *Kudoa* species from eastern Australia: *Kudoa thyrsites* from mahi mahi (*Coryphaena hippurus*), *Kudoa amamiensis* and *Kudoa minithyrsites* n. sp. from sweeper (*Pempheris ypsilychnus*). J Eukaryot Microbiol..

[82] Henderson M, Okamura B (2004). The phylogeography of salmonid proliferative kidney disease in Europe and North America. Proc R Soc Lond B-Biol Sci..

[83] Liu XH, Batueva MD, Zhao YL, Zhang JY, Zhang QQ, Li TT (2016). Morphological and molecular characterisation of *Myxobolus pronini* n. sp. (Myxozoa: Myxobolidae) from the abdominal cavity and visceral serous membranes of the gibel carp *Carassius auratus gibelio* (Bloch) in Russia and China. Parasit Vectors..

[84] Fiala I (2006). The phylogeny of Myxosporea (Myxozoa) based on small subunit ribosomal RNA gene analysis. Int J Parasitol..

[85] Gunter N, Adlard R (2010). The demise of *Leptotheca* Thélohan, 1895 (Myxozoa: Myxosporea: Ceratomyxidae) and assignment of its species to *Ceratomyxa* Thélohan, 1892 (Myxosporea: Ceratomyxidae), *Ellipsomyxa* Køie, 2003 (Myxosporea: Ceratomyxidae), *Myxobolus* Bütschli, 1882 and *Sphaerospora* Thélohan, 1892 (Myxosporea: Sphaerosporidae). Syst Parasitol..

[86] Alama-Bermejo G, Raga JA, Holzer AS (2011). Host-parasite relationship of *Ceratomyxa puntazzi* n. sp. (Myxozoa: Myxosporea) and sharpsnout seabream *Diplodus puntazzo* (Walbaum, 1792) from the Mediterranean with first data on ceratomyxid host specificity in sparids. Vet Parasitol..

[87] de Vienne DM, Refrégier G, López-Villavicencio M, Tellier A, Hood ME, Giraud T (2013). Cospeciation *vs* host-shift speciation: methods for testing, evidence from natural associations and relation to coevolution. New Phytol..

[88] Vega GC, Wiens JJ (2012). Why are there so few fish in the sea?. P Roy Soc Lond B Biol..

[89] Cooper N, Griffin R, Franz M, Omotayo M, Nunn CL (2012). Phylogenetic host specificity and understanding parasite sharing in primates. Ecol Lett..

[90] Huang S, Bininda-Emonds ORP, Stephens PR, Gittleman JL, Altizer S (2014). Phylogenetically related and ecologically similar carnivores harbour similar parasite assemblages. J Anim Ecol..

[91] Reyjol Y, Hugueny B (2007). D. Pont, Bianco PG, Beier U, Caiola N, et al. Patterns in species richness and endemism of European freshwater fish. Global Ecol Biogeogr..

[92] Freyhof J, Brooks E (2011). European Red List of freshwater fishes. Luxembourg: Publications Office of the European Union.

[93] Tedersoo L, Bahram M, Dickie IE (2014). Does host plant richness explain diversity of ectomycorrhizal fungi? Re-evaluation of Gao et al. (2013) data sets reveals sampling effects. Mol Ecol..

[94] Gleeson R, Adlard R (2011). Morphological and genetic analysis of three new species of *Ceratomyxa* Thélohan, 1892 (Myxozoa: Myxosporea) from carcharhinid sharks off Australia. Syst Parasitol..

[95] Gleeson RJ, Adlard RD (2012). Phylogenetic relationships amongst *Chloromyxum* Mingazzini, 1890 (Myxozoa: Myxosporea), and the description of six novel species from Australian elasmobranchs. Parasitol Int..

[96] Kodádková A, Bartošová-Sojková P, Holzer AS, Fiala I (2015). *Bipteria vetusta* n. sp. - an old parasite in an old host: tracing the origin of myxosporean parasitism in vertebrates. Int J Parasitol..

[97] Molnár K (1993). *Sphaerospora siluri* n. sp. (Myxosporea: Sphaerosporidae) in the kidney of the sheatfish (*Silurus glanis*). Acta Vet Hung..

