# Supplementary material for: Biodiversity and host-parasite cophylogeny of Sphaerospora (sensu stricto) (Cnidaria: Myxozoa)
Source: Parasit Vectors. 2018 Jun 15;11:347. doi: 10.1186/s13071-018-2863-z (PMC6002976; doi:10.1186/s13071-018-2863-z)
Supplement: Supplementary file 1 — Table S1. List of fish hosts examined for Sphaerospora spp. infections with information about locality, number of fish examined, parasite prevalence (positive samples in a black box) and estimated age from the total length. (DOCX 51 kb) [file 13071_2018_2863_MOESM1_ESM.docx]

**Additional file 1: Table S1.** List of fish hosts examined for *Sphaerospora* infections with information about locality, number of fish examined, parasite prevalence (positive samples in black box) and estimated age from the total length.

| **Fish species** | **Locality** | **Date of collection** | **Total length (cm)** | **Estimated approx. age (year)** | **No. of fish examined** | **No. of fish infected with *Sphaerospora*** | **Prevalence**  **(%)** |
| --- | --- | --- | --- | --- | --- | --- | --- |
| **Freshwater:** | | | | | | | |
| **Order: Anguilliformes > Family:** **Anguillidae** | | | | | | | |
| *Anguilla anguilla* | Plav, Malše River, CZ | 14.03.2014 | 30 | 3 | 1 | 0 | 0 |
| **Order: Centrarchiformes > Family: Centrarchidae** | | | | | | | |
| *Lepomis gibbosus* | Jindriš fish farm, CZ | 17.10.2012 | 4–10 | 0+ | 4 | 1 (LM+PCR) (SPS) | 25 |
|  |  | 11.06.2013 | 13–14 | 1 | 3 | 0 | 0 |
|  | Oxbow of Dyje River, CZ | 03.09.2012 | 4-7 | 0 | 7 | 0 | 0 |
| **Order: Clupeiform > Family:** **Clupeidae** | | | | | | | |
| *Alosa fallax* | Iseo Lake, IT | 12.03.2013 | 12–17 | 1 | 15 | 0 | 0 |
| **Order: Cypriniformes > Family: Acheilognathidae** | | | | | | | |
| *Rhodeus sericeus amarus* | Jindřiš fish farm, CZ | 17.10.2012 | 6–12 | 0+ | 32 | 0 | 0 |
|  | Oxbow of Dyje River, CZ | 04.09.2012 | 4 | 0+ | 1 | 0 | 0 |
|  | Dyje River, CZ | 24.08.2012 | N/A | N/A | 1 | 0 | 0 |
| **Order: Cypriniformes > Family: Cyprinidae** | | | | | | | |
| *Barbus barbus* | Oxbow of Dyje River, CZ | 04.09.2012 | 10 | 0+ | 1 | 0 | 0 |
|  | Dyje River, CZ | 14.06.2012 | 16.5 | 1 | 1 | 0 | 0 |
|  |  | 24.08.2012 | 10 | 0+ | 1 | 0 | 0 |
| **Order: Cypriniformes > Family:** **Gobionidae** | | | | | | | |
| *Gobio gobio* | Jindřiš fish farm, CZ | 17.10.2012 | 7–11 | 0+ | 5 | 0 | 0 |
|  |  | 11.06.2013 | 7–12 | 0+ | 3 | 1 (LM+PCR) (ESPS) | 33 |
|  | Morava river, CZ | 02.09.2012 | 6–7 | 0+ | 4 | 0 | 0 |
|  | Oslava River, CZ | 05.09.2012 | 5 | 0+ | 1 | 0 | 0 |
|  | Plav, Malše River, CZ | 14.03.2014 | 11–15 | 0–1 | 19 | 0 | 0 |
|  | Dyje River, CZ | 14.06.2012 | 7–10 | 0+ | 10 | 0 | 0 |
|  | Rožmberk Pond, CZ | 12.10.2012 | 8.5 | 0+ | 1 | 0 | 0 |
|  | Svratka River, CZ | 05.09.2012 | 7 | 0+ | 1 | 0 | 0 |
|  | Želivka Dam, CZ | 12.03.2013 | 8 | 0+ | 1 | 0 | 0 |
| **Order: Cypriniformes > Family: Leuciscidae** | | | | | | | |
| *Abramis brama* | Balaton Lake, HU | 21.05.2002 | N/A | 1+ | 1 | 1 (LM+PCR) (SPS) * | 100 |
|  |  | 01.04.2004 | N/A | N/A | 1 | 0 | 0 |
|  | Želivka Dam, CZ | 18.04.2013 | 36 | 3 | 1 | 1 (LM+PCR) (ESPS) | 100 |
|  | Římov water reservoir, CZ | 29.06.2016 | 9–16.5 | 0–1 | 13 | 1 (LM+PCR) (SPS) | 8 |
| *Alburnus alburnus* | Balaton Lake, HU | 24.06.2002 | N/A | 1+ | 1 | 0 | 0 |
|  | Jindřiš fish farm, CZ | 17.10.2012 | 4–6 | 0+ | 46 | 0 | 0 |
|  | Oxbow of Dyje River, CZ | 03.09.2012 | 11–12 | 0+ | 2 | 0 | 0 |
|  | Dyje River, CZ | 14.06.2012 | 9–12 | 0+ | 3 | 0 | 0 |
|  | Rožmberk Pond, CZ | 12.10.2012 | 8–9 | 0+ | 2 | 0 | 0 |
|  | Horní Hluboký Pond, Strmilov, CZ | 19.05.2013 | 6 | 0+ | 1 | 0 | 0 |
| *Aspius aspius* | Jindřiš fish farm, CZ | 17.10.2012 | 4–8 | 0+ | 12 | 0 | 0 |
|  | Oslava River, CZ | 05.09.2012 | 9 | 0+ | 1 | 0 | 0 |
|  | Oxbow of Dyje River, CZ | 04.09.2012 | 7–14 | 0+ | 2 | 0 | 0 |
|  | Dyje River, CZ | 14.06.2012 | 10–12 | 0+ | 5 | 0 | 0 |
|  |  | 24.08.2012 | 8–12 | 0+ | 3 | 0 | 0 |
|  | Svratka River, CZ | 05.09.2012 | 8 | 0+ | 1 | 0 | 0 |
| *Blicca bjoerkna* | Balaton, HU | 16.04.2004 | N/A | 1+ | 1 | 1 (LM+PCR) (SPS) | 100 |
|  | Oxbow of Dyje River, CZ | 03.09.2012 | 7–8 | 0+ | 3 | 0 | 0 |
|  |  | 04.09.2012 | 7–14 | 0+ | 2 | 0 | 0 |
|  | Plav, Malše River, CZ | 14.03.2014 | 13 | 0+ | 1 | 0 | 0 |
|  | Rožmberk Pond, CZ | 12.10.2012 | 10–24 | 0–1 | 5 | 0 | 0 |
|  | Dyje River, CZ | 14.06.2012 | 9–10 | 0+ | 2 | 0 | 0 |
|  |  | 24.08.2012 | 7–10 | 0+ | 18 | 0 | 0 |
| *Chondrostoma nasus* | Oslava River, CZ | 05.09.2012 | 9 | 0+ | 1 | 0 | 0 |
|  | Plav, Malše River, CZ | 14.03.2014 | 26.5 |  | 1 | 0 | 0 |
| *Leucaspius delineatus* | Jindřiš fish farm, CZ | 17.10.2012 | 5–6 | 0+ | 2 | 0 | 0 |
| *Leuciscus idus* | Oslava River, CZ | 05.09.2012 | 8 | 0+ | 1 | 0 | 0 |
|  | Oxbow of Dyje River, CZ | 03.09.2012 | 10–16 | 0–1 | 7 | 0 | 0 |
|  |  | 04.09.2012 | 7–10 | 0+ | 5 | 0 | 0 |
|  | Dyje River, CZ | 14.06.2012 | 12–15 | 0–1 | 3 | 0 | 0 |
|  |  | 24.08.2012 | 6–8 | 0+ | 3 | 1 (LM+PCR) (ESPS) | 33 |
| *Leuciscus leuciscus* | Plav, Malše River, CZ | 14.03.2014 | 7–26 | 0-1 | 27 | 1 (LM+PCR) (SPS) | 4 |
|  | Oslava River, CZ | 05.09.2012 | 5–6 | 0+ | 2 | 1 (LM+PCR) (ESPS) | 50 |
|  | Dyje River, CZ | 14.03.2012 | N/A | N/A | 1 | 0 | 0 |
| *Rutilus rutilus* | Jindřiš fish farm, CZ | 08.07.2014 | 17–20 | 1 | 2 | 0 | 0 |
|  | Oxbow of Dyje River, CZ | 03.09.2012 | 6–14 | 0+ | 6 | 0 | 0 |
|  |  | 04.09.2012 | 9–14 | 0+ | 5 | 0 | 0 |
|  |  | 05.09.2012 | 10 | 0+ | 1 | 0 | 0 |
|  | Oslava River, CZ | 05.09.2012 | 7–12 | 0+ | 3 | 0 | 0 |
|  | Plav, Malše River, CZ | 14.03.2014 | 13–20 | 1 | 19 | 0 | 0 |
|  | Dyje River, CZ | 14.06.2012 | 7–13 | 0+ | 7 | 1 (PCR) | 14 |
|  |  | 24.08.2012 | 6–11 | 0+ | 3 | 0 | 0 |
|  | Rájský Pond, CZ | 26.03.2017 | 13–15 | 1 | 12 | 5 (LM+PCR) (SPS) | 42 |
|  | Rožmberk Pond, CZ | 12.10.2012 | 8 | 0+ | 2 | 0 | 0 |
|  | Svratka River, CZ | 05.09.2012 | 6–8 | 0+ | 6 | 0 | 0 |
| *Scardinius erythrophthalmus* | Balaton lake, HU | 03.05.2004 | N/A | 1+ | 1 | 1 (LM+PCR) (SPS) * | 100 |
|  | Jindřiš fish farm, CZ | 11.06.2013 | 15–19 | 1 | 4 | 0 | 0 |
|  |  | 08.07.2014 | 16–22 | 1 | 8 | 1 (LM+PCR) (SPS) | 13 |
|  | Jihlava, CZ | 28.08.2012 | 10 | 0+ | 1 | 0 | 0 |
|  | Horní Hluboký Pond, Strmilov, CZ | 09.10.2013 | 17.5 | 1 | 1 | 0 | 0 |
|  |  | 16.04.2014 | 17.5 | 1 | 1 | 0 | 0 |
|  | Želivka Dam, CZ | 06.04.2017 | 14 | 0+ | 1 | 1 (LM+PCR) (SPS) | 100 |
| *Squalius cephalus* | Plav, Malše River, CZ | 14.03.2014 | 12–25 | 0–1 | 34 | 1 (LM+PCR) (ESPS) | 3 |
|  | Oslava River, CZ | 05.09.2012 | 7–8 | 0+ | 15 | 0 | 0 |
|  | Oxbow of Dyje River, CZ | 04.09.2012 | 6–7 | 0+ | 3 | 0 | 0 |
|  |  | 05.09.2012 | 6–7 | 0+ | 3 | 0 | 0 |
|  | Dyje River, CZ | 14.06.2012 | 10–16 | 0–1 | 4 | 1 (LM+PCR) (SPS) | 25 |
|  | Svratka River, CZ | 05.09.2012 | 5–7 | 0+ | 2 | 0 | 0 |
| **Order: Cypriniformes > Family:** **Tincidae** | | | | | | | |
| *Tinca tinca* | Jindřiš fish farm, CZ | 17.10.2012 | 5–7 | 0+ | 3 | 0 | 0 |
|  | Jihlava, CZ | 28.08.2012 | 18 | 1 | 1 | 0 | 0 |
|  | Horní Hluboký Pond, Strmilov, CZ | 17.05.2013 | 12 | 0+ | 1 | 0 | 0 |
|  |  | 19.05.2013 | 14 | 0+ | 1 | 0 | 0 |
|  |  | 11.06.2013 | 16–19 | 1 | 6 | 0 | 0 |
|  |  | 26.07.2013 | 11–17 | 1 | 3 | 0 | 0 |
| **Order: Cypriniformes > Family: Xenocyprididae** | | | | | | | |
| *Ctenopharyngodon idella* | Massa Finalese, IT | 13.10.2011 | N/A | N/A | 1 | 1 (LM+PCR) (SPS) * | 100 |
|  | Horní Hluboký Pond, Strmilov, CZ | 17.05.2013 | 9 | 0+ | 1 | 0 | 0 |
| **Order: Gadiformes > Family:** **Gadidae** | | | | | | | |
| *Lota lota* | Iseo Lake, IT | 05.02.2013 | 36-38 | 2-3 | 2 | 2 (PCR) | 100 |
|  | Oxbow of Dyje River, CZ | 03.09.2012 | 20 | 1-2 | 1 | 0 | 0 |
| **Order: Gobiiformes > Family:** **Gobiidae** | | | | | | | |
| *Proterorhinus semilunaris* | Morava River, CZ | 02.09.2012 | 6–7 | 0+ | 3 | 0 | 0 |
| **Order:** **Perciformes > Family: Percidae** | | | | | | | |
| *Gymnocephalus cernua* | Horní Hluboký Pond, Strmilov, CZ | 17.05.2013 | 13.5 | 0+ | 1 | 0 | 0 |
|  | Želivka Dam, CZ | 12.03.2013 | 15 | 0–1 | 1 | 0 | 0 |
| *Perca fluviatilis* | Iseo Lake, IT | 12.03.2013 | 14–17 | 1 | 34 | 0 | 0 |
|  | Plav, Malše River, CZ | 14.03.2014 | 13–14 | 0–1 | 3 | 0 | 0 |
|  | Rožmberk Pond, CZ | 12.10.2012 | 8–9 | 0+ | 2 | 0 | 0 |
|  | Dyje River, CZ | 24.08.2012 | 8–9 | 0+ | 3 | 0 | 0 |
|  | Horní Hluboký Pond, Strmilov, CZ | 09.10.2013 | 18–20 | 1 | 8 | 0 | 0 |
| *Sander lucioperca* | Rožmberk Pond, CZ | 12.10.2012 | 8–9 | 0+ | 30 | 1 (PCR) | 3 |
| **Order: Siluriformes > Family: Siluridae** | | | | | | | |
| *Silurus glanis* | Milada Lake, CZ | 31.03.2017 | 19 | 1 | 1 | 1 (PCR) | 100 |
| **Marine:** | | |  |  | | | |
| **Order: Beloniformes > Family:** **Belonidae** | | | | | | | |
| *Strongylura notata* | Tidy Island, USA | 01.11.2012 | 28–36 | 1–2 | 2 | 0 | 0 |
| **Order: Elopiformes > Family: Elopidae** | | | | | | | |
| *Elops saurus* | Tidy Island, USA | 01.11.2012 | 32 | 2–3 | 1 | 1 (LM+PCR) (SPS) | 100 |
| **Order: Salmoniformes > Family:** **Salmonidae** | | | | | | | |
| *Oncorhynchus mykiss* | Jindřiš fish farm, CZ | 08.07.2014 | 25–27 | 1–2 | 4 | 0 | 0 |

Abbreviations: LM = samples positive by light microscopy, PCR = samples positive by PCR, SPS = sporogonic stage containing mature spores, ESPS = early sporogonic stage (no mature spores), * = spore measurement not available, CZ = Czech Republic, HU = Hungary, IT = Italy and N/A = data not available.
