# Supplementary material for: Biodiversity and host-parasite cophylogeny of Sphaerospora (sensu stricto) (Cnidaria: Myxozoa)
Source: Parasit Vectors. 2018 Jun 15;11:347. doi: 10.1186/s13071-018-2863-z (PMC6002976; doi:10.1186/s13071-018-2863-z)
Supplement: Supplementary file 2 — Table S2. List of PCR primer combinations and other PCR details applied to amplify 18S rDNA sequences of different sphaerosporids. (DOCX 50 kb) [file 13071_2018_2863_MOESM2_ESM.docx]

**Additional file 2: Table S2.** List of PCR primer combinations and other PCR details applied to amplify 18S rDNA sequences of different sphaerosporids.

| **Species** | **Primers combination** | **PCR type/Taq** | **Tm (°C)** | **Product size (bp)** | **Reference** |
| --- | --- | --- | --- | --- | --- |
| *Sphaerospora* sp. ex *Abramis brama* | Part 1: Erib1-Erib10  Erib1- SphSSUR680 | Nested/T | 60 | NI | Barta et al. [1] |
|  |  |  | 55 | 700 | Holzer et al. [2] |
|  | Part 2: Erib1- SelegSSU2960R  MyxGP2F- SelegSSU2750R | Nested/T | 50 | NI | Bartošová et al. [3] |
|  |  |  | 50 | 1600 |  |
|  | Part 3: Erib1-Erib10  MyxGP2F- Act1R | Nested/T | 60 | NI | Barta et al. [1] |
|  |  |  | 58 | 1800 | Holzer et al. [2, 4] |
|  | Part 4: Erib1-Erib10  SphFWSSU1243F-SphFWSSU3418R | Nested/T | 60 | NI | Barta et al. [1] |
|  |  |  | 56 | 2000 | Present study |
|  | Part 5: PsSSU1850F-Erib10  PsSSU2110F-Erib10 | Nested/T | 55 | NI | Bartošová et al. [3] |
|  |  |  | 55 | 1000 |  |
| *Sphaerospora* a*brami* n. sp. | Part 1: Erib1- SelegSSU2960R  MyxGP2F- SelegSSU2750R | Nested/T | 50 | NI | Bartošová et al. [3] |
|  |  |  | 50 | 1600 |  |
|  | Part 2: Erib1-Erib10  SphFWSSU1243F-SphFWSSU3418R | Nested/T | 60 | NI | Barta et al. [1] |
|  |  |  | 56 | 2000 | Present study |
|  | Part 3: PsSSU1850F-Erib10  PsSSU2110F-Erib10 | Nested/T | 55 | NI | Bartošová et al. [3] |
|  |  |  | 55 | 1000 |  |
| *Sphaerospora* *bliccae* n. sp. | Part 1: Erib1- SelegSSU2960R  MyxGP2F- SelegSSU2750R | Nested/T | 50 | NI | Bartošová et al. [3] |
|  |  |  | 50 | 1600 |  |
|  | Part 2: Erib1-Erib10  SphFWSSU1243F-SphFWSSU3418R | Nested/T | 60 | NI | Barta et al. [1] |
|  |  |  | 56 | 2000 | Present study |
|  | Part 3: PsSSU1850F-Erib10  PsSSU2110F-Erib10 | Nested/T | 55 | NI | Bartošová et al. [3] |
|  |  |  | 55 | 1000 |  |
| *Sphaerospora* sp. ex *Ctenopharyngodon idella* | Part 1: Erib1-Erib10  Erib1- SphSSUR680 | Nested/T | 60 | NI | Barta et al. [1] |
|  |  |  | 55 | 700 | Holzer et al. [2] |
|  | Part 2: Erib1-Erib10  SphFWSSU1243F-SphFWSSU3418R | Nested/T | 60 | NI | Barta et al. [1] |
|  |  |  | 56 | 2000 | Present study |
|  | Part 3: Ameb1200F – Erib10 | Nested/T | 50 | 12000 | Dyková et al. [5] |
|  | Part 4: SphGoldSSU2F2450-Erib10  SphGoldSSU2F2483^-^ Erib10 | Nested/P | 48 | NI | Bartošová et al. [3] |
|  |  |  | 49 | 1200 |  |
| *Sphaerospora dentata* n. sp. | Part 1: Erib1-Erib10  Erib1- SphSSUR680 | Nested/T | 60 | NI | Barta et al. [1] |
|  |  |  | 55 | 700 | Holzer et al. [2] |
|  | Part 2: Erib1-Erib10  MyxGP2F- Act1R | Nested/T | 60 | NI | Barta et al. [1] |
|  |  |  | 58 | 1900 | Holzer et al. [2, 4] |
|  | Part 3: Erib1- SelegSSU2960R  MyxGP2F- SelegSSU2750R | Nested/T | 50 | NI | Bartošová et al. [3] |
|  |  |  | 50 | 1600 |  |
|  | Part 4: Erib1-Erib10  SphFWSSU1243F-SphFWSSU3418R | Nested/T | 60 | NI | Barta et al. [1] |
|  |  |  | 56 | 2000 | Present study |
|  | Part 5: PsSSU1850F-Erib10  PsSSU2110F-Erib10 | Nested/T | 55 | NI | Bartošová et al. [3] |
|  |  |  | 55 | 1000 |  |
| *Sphaerospora diminuta* | Part 1: Erib1-SphLgibSSU286R | Non-nested/P | 62 | 1000 | Present study |
|  | Part 2: Erib1-Erib10  MyxGP2F- Act1R | Nested/T | 60 | NI | Barta et al. [1] |
|  |  |  | 58 | 1400 | Holzer et al. [2, 4] |
|  | Part 3: SphLgibSSU483F-Erib10 | Non-nested/P | 66 | 1500 | Present study |
| *Sphaerospora diversa* n. sp. (*Leuciscus idus*) | Part 1: Erib1-SphleuSSUR | Non-nested/P | 60 | 1000 | Present study |
|  | Part 2: SphleuSSUF-Erib10 | Non-nested/P | 60 | 2000 | Present study |
|  | Part 3: Erib1-Erib10  MyxGP2F- Act1R | Nested/T | 60 | NI | Barta et al. [1] |
|  |  |  | 58 | 1800 | Holzer et al. [2, 4] |
|  | Part 4: Erib1-Erib10  SphFWSSU1243F-SphFWSSU3418R | Nested/T | 60 | NI | Barta et al. [1] |
|  |  |  | 56 | 2000 | Present study |
| *Sphaerospora diversa* n. sp. (*Leuciscus leuciscus*) | Part 1: Erib1-Erib10  Erib1- SphSSUR680 | Nested/T | 60 | NI | Barta et al. [1] |
|  |  |  | 55 | 700 | Holzer et al. [2] |
|  | Part 2: Erib1- SelegSSU2960R  MyxGP2F- SelegSSU2750R | Nested/T | 50 | NI | Bartošová et al. [3] |
|  |  |  | 50 | 1600 |  |
|  | Part 3: Erib1-Erib10  SphFWSSU1243F-SphFWSSU3418R | Nested/T | 60 | NI | Barta et al. [1] |
|  |  |  | 56 | 2000 | Present study |
|  | Part 4: PsSSU1850F-Erib10  PsSSU2110F-Erib10 | Nested/T | 55 | NI | Bartošová et al. [3] |
|  |  |  | 55 | 1000 |  |
| *Sphaerospora diversa* n. sp. (*Squalius cephalus*) | Part 1: Erib1-Erib10  Erib1- SphSSUR680 | Nested/T | 60 | NI | Barta et al. [1] |
|  |  |  | 55 | 700 | Holzer et al. [2] |
|  | Part 2: Erib1- SelegSSU2960R  MyxGP2F- SelegSSU2750R | Nested/T | 50 | NI | Bartošová et al. [3] |
|  |  |  | 50 | 1600 |  |
|  | Part 3: Erib1-Erib10  SphFWSSU1243F-SphFWSSU3418R | Nested/T | 60 | NI | Barta et al. [1] |
|  |  |  | 56 | 2000 | Present study |
|  | Part 4: PsSSU1850F-Erib10  PsSSU2110F-Erib10 | Nested/T | 55 | NI | Bartošová et al. [3] |
|  |  |  | 55 | 1000 |  |
| *Sphaerospora* *elopi* n. sp. | Part 1: SphElopsSSU615F-Erib10 | Non-nested/P | 60 | 1200 | Present study |
| *Sphaerospora* sp. ex *Gobio gobio* | Part 1: Erib1-Erib10  SphFWSSU1243F-SphFWSSU3418R | Nested/T | 60 | NI | Barta et al. [1] |
|  |  |  | 56 | 2000 | Present study |
| *Sphaerospora gutta* n. sp. | Part 1: Erib1-Erib10  Erib1- SphSSUR680 | Nested/T | 60 | NI | Barta et al. [1] |
|  |  |  | 55 | 700 | Holzer et al. [2] |
|  | Part 2: Erib1-Erib10  MyxGP2F- Act1R | Nested/T | 60 | NI | Barta et al. [1] |
|  |  |  | 58 | 1900 | Holzer et al. [2, 4] |
|  | Part 3: Erib1-Erib10  SphFWSSU1243F-SphFWSSU3418R | Nested/T | 60 | NI | Barta et al. [1] |
|  |  |  | 56 | 2000 | Present study |
|  | Part 4: PsSSU1850F-Erib10  PsSSU2110F-Erib10 | Nested/T | 55 | NI | Bartošová et al. [3] |
|  |  |  | 55 | 1000 |  |
| *Sphaerospora* sp. ex *Lota lota* | Part 1: Erib1-Erib10  Erib1-BasalSphCladeSSU1310R | Nested/T | 60 | NI | Barta et al. [1] |
|  |  |  | 57 | 1200 | Bartošová et al. [3] |
|  | Part 2: Erib1-Erib10  MyxGP2F- Act1R | Nested/T | 60 | NI | Barta et al. [1] |
|  |  |  | 58 | 1400 | Holzer et al. [2, 4] |
|  | Part 3: Erib1- SelegSSU2960R  MyxGP2F- SelegSSU2750R | Nested/T | 50 | NI | Bartošová et al. [3] |
|  |  |  | 50 | 1600 |  |
|  | Part 4: Erib1-Erib10  SphFWSSU1243F-SphFWSSU3418R | Nested/T | 60 | NI | Barta et al. [1] |
|  |  |  | 56 | 2000 | Present study |
|  | Part 5: PsSSU1850F-Erib10  PsSSU2110F-Erib10 | Nested/T | 55 | NI | Bartošová et al. [3] |
|  |  |  | 55 | 1000 |  |
| *Sphaerospora rutili* n. sp. | Part 1: Erib1-Erib10  Erib1- SphSSUR680 | Nested/T | 60 | NI | Barta et al. [1] |
|  |  |  | 55 | 700 | Holzer et al. [2] |
|  | Part 2: Erib1- SelegSSU2960R  MyxGP2F- SelegSSU2750R | Nested/T | 50 | NI | Bartošová et al. [3] |
|  |  |  | 50 | 1600 |  |
|  | Part 3: Erib1-Erib10  SphFWSSU1243F-SphFWSSU3418R | Nested/T | 60 | NI | Barta et al. [1] |
|  |  |  | 56 | 2000 | Present study |
| *Sphaerospora* sp. ex *Rutilus rutilus* | Part 1: Erib1-Erib10  SphFWSSU1243F-SphFWSSU3418R | Nested/T | 60 | NI | Barta et al. [1] |
|  |  |  | 56 | 2000 | Present study |
| *Sphaerospora* sp. ex *Sander lucioperca* | Part 1: Erib1-Erib10  Erib1-BasalSphCladeSSU1310R | Nested/T | 60 | NI | Barta et al. [1] |
|  |  |  | 57 | 1200 | Bartošová et al. [3] |
|  | Part 2: Erib1- SelegSSU2960R  MyxGP2F- SelegSSU2750R | Nested/T | 50 | NI | Bartošová et al. [3] |
|  |  |  | 50 | 1600 |  |
|  | Part 3: Erib1-Erib10  SphFWSSU1243F-SphFWSSU3418R | Nested/T | 60 | NI | Barta et al. [1] |
|  |  |  | 56 | 2000 | Present study |
|  | Part 4: PsSSU1850F-Erib10  PsSSU2110F-Erib10 | Nested/T | 55 | NI | Bartošová et al. [3] |
|  |  |  | 55 | 1000 |  |
| *Sphaerospora* sp. *Scardinius* *erythrophthalmus* | Part 1: Erib1-Erib10  SphFWSSU1243F-SphFWSSU3418R | Nested/T | 60 | NI | Barta et al. [1] |
|  |  |  | 56 | 2000 | Present study |
|  | Part 2: PsSSU1850F-Erib10  PsSSU2110F-Erib10 | Nested/T | 55 | NI | Bartošová et al. [3] |
|  |  |  | 55 | 1000 |  |
| *Sphaerospora* sp. ex *Silurus glanis* | Part 1: Erib1-Erib10  MyxGP2F- Act1R | Nested/T | 60 | NI | Barta et al. [1] |
|  |  |  | 58 | 1800 | Holzer et al. [2, 4] |
|  | Part 2: Erib1-Erib10  SphFWSSU1243F-SphFWSSU3418R | Nested/T | 60 | NI | Barta et al. [1] |
|  |  |  | 56 | 2000 | Present study |
|  | Part 3: PsSSU1850F-Erib10  PsSSU2110F-Erib10 | Nested/T | 55 | NI | Bartošová et al. [3] |
|  |  |  | 55 | 1000 |  |
| *Sphaerospora squalii* n. sp. | Part 1: Erib1-Erib10  Erib1- SphSSUR680 | Nested/T | 60 | NI | Barta et al. [1] |
|  |  |  | 55 | 700 | Holzer et al. [2] |
|  | Part 2: Erib1-Erib10  MyxGP2F- Act1R | Nested/T | 60 | NI | Barta et al. [1] |
|  |  |  | 58 | 2000 | Holzer et al. [2, 4] |
|  | Part 3: Erib1-Erib10  SphFWSSU1243F-SphFWSSU3418R | Nested/T | 60 | NI | Barta et al. [1] |
|  |  |  | 56 | 2000 | Present study |

Abbreviations: P: Taq-Purple DNA polymerase, T = TITANIUM Taq polymerase, Tm = annealing temperature, NI = not identified.

**References**

1. Barta JR, Martin DS, Liberator PA, Dashkewitz M, Anderson JW, Deighner SD, et al. Phylogenetic relationships among eight *Eimeria* species infecting domestic fowl inferred using complete small subunit ribosomal DNA sequences. J Parasitol. 1997;83: 262–271
2. Holzer AS, Bartošová P, Pecková H, Tyml T, Atkinson S, Bartholomew J, et al. Who's who' in renal sphaerosporids (Bivalvulida: Myxozoa) from common carp, Prussian carp and goldfish - molecular identification of cryptic species, blood stages and new members of *Sphaerospora sensu stricto*. Parasitology. 2013;140:46-60
3. Bartošová P, Fiala I, Jirků M, Cinková M, Caffara M, Fioravanti ML, et al. *Sphaerospora sensu stricto*: Taxonomy, diversity and evolution of a unique lineage of myxosporeans (Myxozoa). Mol Phylogenet Evol. 2013;68:93-105
4. Holzer AS, Sommerville C, Wootten R. Molecular relationships and phylogeny in a community of myxosporeans and actinosporeans based on their 18S rDNA sequences. Int J Parasitol. 2004;34:1099-1111
5. Dyková I, Pecková H, Kostka M. Introduction of *Mayorella gemmifera* Schaeffer, 1926 into Phylogenetic Studies of Amoebozoa. Acta Protozool. 2008;47: 205–210
