# Supplementary material for: Biodiversity and host-parasite cophylogeny of Sphaerospora (sensu stricto) (Cnidaria: Myxozoa)
Source: Parasit Vectors. 2018 Jun 15;11:347. doi: 10.1186/s13071-018-2863-z (PMC6002976; doi:10.1186/s13071-018-2863-z)
Supplement: Supplementary file 3 — Table S3. Newly designed primers used in this study. (DOCX 28 kb) [file 13071_2018_2863_MOESM3_ESM.docx]

**Additional file 3: Table S3.** Newly designed primers used in this study.

| **18S rDNA Primer** | **Sequence (5´–3´)** |
| --- | --- |
| SphFWSSU1243F | GTCCAATTGCTTGAACCACCC |
| SphFWSSU3418R | TGATGCAACTTGGACAGGCTC |
| SphElopsSSU615F | TGGGCATGCGTGCGTATGTG |
| SphLgibSSU286R | CAATCAAGTGTCCCAGCCTCC |
| SphLgibSSU483F | GATACAAAAGGGCCGGGCAG |
| SphleuSSUF | AGTTACGCACTCTGGCGTGTG |
| SphleuSSUR | GATCTGTGTGCGCATACCGTC |
