# Supplementary material for: Biodiversity and host-parasite cophylogeny of Sphaerospora (sensu stricto) (Cnidaria: Myxozoa)
Source: Parasit Vectors. 2018 Jun 15;11:347. doi: 10.1186/s13071-018-2863-z (PMC6002976; doi:10.1186/s13071-018-2863-z)
Supplement: Supplementary file 4 — Table S4. PCR cycling parameters used for sphaerosporid amplification. (DOCX 28 kb) [file 13071_2018_2863_MOESM4_ESM.docx]

**Additional file 4: Table S4.** PCR cycling parameters used for sphaerosporid amplification.

| **PCR reaction** | **For Taq-purple** | **For TITANIUM taq** |
| --- | --- | --- |
| Denaturation of DNA | 95°C (3 min) | 95°C (3min) |
| 35 cycles of amplification | 94°C (1 min) | 94°C (1 min) |
|  | Annealing temperature (1 min) | Annealing temperature (1 min) |
|  | 72°C # | 68°C # |
| Extension | 72°C (5 min) | 68°C (5 min) |

# = time varied according to the PCR product length. Generally, 1 min of extension step used for 1000 bp long product.
