# Supplementary material for: Biodiversity and host-parasite cophylogeny of Sphaerospora (sensu stricto) (Cnidaria: Myxozoa)
Source: Parasit Vectors. 2018 Jun 15;11:347. doi: 10.1186/s13071-018-2863-z (PMC6002976; doi:10.1186/s13071-018-2863-z)
Supplement: Supplementary file 6 — Table S5. The lengths of variable regions (base pairs) and GC-content (percentage; in parenthesis) of newly obtained 18S rDNA Sphaerospora sequences (bold numbers indicate the highest values). (DOCX 32 kb) [file 13071_2018_2863_MOESM6_ESM.docx]

**Additional file 6: Table S5.** The lengths of variable regions (base pairs) and GC-content (percentage; in parenthesis) of newly obtained 18S rDNA *Sphaerospora* sequences (bold numbers indicate the highest values).

| ***Sphaerospora* species** | | **Length of variable regions** | | | | | | | | | | **Length of 18S rDNA** |
| --- | --- | --- | --- | --- | --- | --- | --- | --- | --- | --- | --- | --- |
|  |  | **V1** | **V2** | **V3** | **V4** | | | **V5** | **V7** | **V8** | **V9** |  |
|  |  |  |  |  | **E23_ 1–7** | **E23_ 13–15** | **total** |  |  |  |  |  |
| *Sphaerospora* sp. ex *Abramis brama* | | 33 (42) | **315** (52) | 61 (48) | 534 | 566 | 1181 (52) | 65 (45) | 406 (53) | 59 (48) | - | 3162 (51) |
| *Sphaerospora abrami* n. sp. | | - | - | 61 (48) | 535 | 661 | **1277** (51) | 65 (45) | 407 (52) | 59 (48) | - | 3100 (50) |
| *Sphaerospora bliccae* n. sp. | | - | ^-^ | 61 (44) | 396 | 664 | 1141 (53) | 61 (48) | **444** (54) | 61 (49) | **-** | 3016 (50) |
| *Sphaerospora* sp. ex *Ctenopharyngodon idella* | | 33 (39) | 272 (52) | 61 (49) | 409 | 765 | 1255 (57) | 80 (56) | **448** (58) | 57 (49) | - | 3306 (51) |
| *Sphaerospora dentata* n. sp. | | 33 (42) | 299 (52) | 61 (48) | 506 | 546 | 1133 (52) | 65 (45) | 405 (52) | 59 (48) | **-** | 3105 (51) |
| *Sphaerospora diminuta* | | 33 (**52**) | 257 (**61**) | 61 (**64**) | 184 | 553 | 844 (65) | **102** (**73**) | 264 (**68**) | 67 (**54**) | - | 2665 (60) |
| *Sphaerospora diversa n. sp.* | *Leuciscus idus* | 33 (42) | 308 (54) | 61 (48) | 454 | 513 | 1048 (53) | 64 (45) | 405 (53) | 59 (48) | 101 (47) | 3049 (52) |
|  | *Leuciscus leuciscus* | 33 (42) | 308 (54) | 61 (48) | 454 | 511 | 1046 (53) | 65 (48) | 405 (53) | 59 (49) | 101 (48) | 3091 (52) |
|  | *Squalius cephalus* | 33 (42) | 308 (54) | 61 (48) | 454 | 511 | 1046 (53) | 65 (48) | 405 (53) | 59 (49) | 101 (47) | 3097 (52) |
| *Sphaerospora* sp. ex *Gobio gobio* | | - | - | - | - | - | - | 65 (45) | 422 (54) | 59 (48) | - | 1196 (52) |
| *Sphaerospora gutta* n. sp. | | 33 (42) | 304 (52) | 61 (48) | 526 | 682 | **1289** (52) | 64 (45) | 408 (53) | 59 (48) | - | 3306 (51) |
| *Sphaerospora* sp. ex *Lota lota* | | 33 (46) | 258 (47) | 60 (47) | 296 | 473 | 845 (46) | 54 (46) | 253 (49) | 57 (49) | - | 2610 (48) |
| *Sphaerospora* sp. ex *Rutilus rutilus* | | - | - | - | - | - | - | - | 406 (51) | 62 (45) | - | 926 (51) |
| *Sphaerospora rutili* n. sp. | | 33 (42) | 299 (51) | 60 (48) | 522 | 542 | 1145 (51) | 65 (45) | 506 (51) | 59 (47) | - | 3150 (50) |
| *Sphaerospora* sp. ex *Sander lucioperca* | | 33 (46) | 310 (54) | 61 (48) | 333 | 610 | 1018 (59) | 54 (46) | 258 (55) | 57 (49) | - | 2847 (54) |
| *Sphaerospora* sp. ex *Scardinius erythrophthalmus* | | - | - | - | - | - | - | 61 (48) | **444** (53) | 61 (49) | - | 1932 (52) |
| *Sphaerospora squalii* n. sp. | | 33 (42) | **316** (51) | 61 (48) | 546 | 547 | 1174 (52) | 65 (45) | 406 (52) | 59 (48) | - | 3173 (51) |
| *Sphaerospora* sp. ex *Silurus glanis* | | - | - | 61 (41) | 294 | 474 | 849 (59) | 63 (48) | 401 (61) | 56 (45) | - | 2548 (55) |
| *Sphaerospora elopi* n. sp.^M^ | | - | - | - | - | - | - | - | - | 57 (49) | - | 429 (51) |

Abbreviations: V = variable region, E23_x = V4 expansion segments and ^M^ = the only sphaerosporid species from the marine I clade.
