# Supplementary material for: Biodiversity and host-parasite cophylogeny of Sphaerospora (sensu stricto) (Cnidaria: Myxozoa)
Source: Parasit Vectors. 2018 Jun 15;11:347. doi: 10.1186/s13071-018-2863-z (PMC6002976; doi:10.1186/s13071-018-2863-z)
Supplement: Supplementary file 9 — Table S8. List of members of Sphaerospora (s.s.) clade (18S rDNA), their vertebrate hosts (complete mitogenome) and GenBank accession numbers (18S rDNA for sphaerosporids and complete mitogenome for vertebrate host) used for cophylogeny studies (CoRe-Pa and Parafit analysis). (DOCX 33 kb) [file 13071_2018_2863_MOESM9_ESM.docx]

**Additional file 9: Table S8**. List of members of *Sphaerospora s. str*. clade (18S rDNA), their vertebrate hosts (complete mitogenome) and GenBank accession numbers (18S rDNA for sphaerosporids and complete mitogenome for vertebrate host) used for cophylogeny studies (CoRe-Pa and Parafit analysis).

| ***Sphaerospora* species name** | **Acc. no. of 18S rDNA** | **Vertebrate host** | **Acc. no. of mitogenome** |
| --- | --- | --- | --- |
| *Sphaerospora angulata* | JQ801527 | *Carassius auratus* | KJ874430 |
| *Sphaerospora dykovae* | JQ801533 | *Cyprinus carpio* | KU050703 |
| *Sphaerospora elegans* | JX286618 | *Gasterosteus aculeatus* | AP002944 |
| *Sphaerospora epinepheli* | HQ871152 | *Epinephelus malabaricus* | NC_028406 |
| *Sphaerospora formosa* | FJ790308 | *Merlangius merlangus* | DQ020496 |
| *Sphaerospora fugu* | AB195805 | *Takifugu rubripes* | NC_004299 |
| *Sphaerospora molnari* | JX431511 | *Cyprinus carpio* | KU050703 |
| *Sphaerospora motemarini* | KC526873 | ***Lutjanus peru*** | KR362299 |
| *Sphaerospora ohlmacheri* | JX286619 | *Rana catesbeiana* | NC_022696 |
| *Sphaerospora ranae* | EF211975 | ***Rana catesbeiana*** | NC_022696 |
| *Sphaerospora sparidarum* | JX286620 | *Sparus aurata* | LK022698 |
| *Sphaerospora sparis* | JX286624 | *Sparus aurata* | LK022698 |
| *Sphaerospora truttae* | AJ581915 | *Salmo trutta* | NC_024032 |
| *Sphaerospora* sp. ex *Liza ramado* | JX286626 | ***Liza affinis*** | KM925142 |
| *Sphaerospora* sp. ex *Chelon labrosus* | JX286625 | *Chelon labrosus* | JF911706 |
| *Sphaerospora* sp. ex *Pomoxis nigromaculatus* | JX286621 | *Pomoxis nigromaculatus* | NC_028298 |
| *Sphaerospora* sp. ex *Ptychadena anchietae* | JX286622 | ***Ptychadena mascareniensis*** | JX564890 |
| *Sphaerospora abrami* n. sp. | MG214664 | *Abramis brama* | NC_020356 |
| *Sphaerospora* sp. ex *Abramis brama* | KY851765 | *Abramis brama* | NC_020356 |
| *Sphaerospora bliccae* n. sp. | KY851767 | *Blicca bjoerkna* | NC_020355 |
| *Sphaerospora* sp. ex *Ctenopharyngodo idella* | KY851768 | *Ctenopharyngodon idella* | EU391390 |
| *Sphaerospora diminuta* | KY851771 | *Lepomis gibbosus* | NC_028284 |
| *Sphaerospora dentata* n. sp. | MG214666 | *Scardinius erythrophthalmus* | NC_031561 |
| *Sphaerospora diversa* n. sp. (*Leuciscus idus*) | KY851774 | *Leuciscus idus* | KF913024 |
| *Sphaerospora diversa* n. sp. (*Leuciscus leuciscus*) | KY851772 | ***Leuciscus idus*** | KF913024 |
| *Sphaerospora diversa* n. sp. (*Squalius cephalus*) | KY851773 | *Squalius cephalus* | AP011214 |
| *Sphaerospora gutta* n. sp. | KY851778 | *Scardinius erythrophthalmus* | NC_031561 |
| *Sphaerospora* sp. ex *Lota lota* | KY851775 | *Lota lota* | KC844053 |
| *Sphaerospora* sp. ex *Sander lucioperca* | KY851777 | *Sander lucioperca* | KM410087 |
| *Sphaerospora* sp. ex *Silurus glanis* | MG214665 | *Silurus glanis* | NC_014261 |
| *Sphaerospora squalii* n. sp. | KY851780 | *Squalius cephalus* | AP011214 |

Bold names are the host substitutions by the closely related species from same genus due to unavailability of the mitogenome of the particular host. For the similar reason, *Sphaerospora olsoni*, *Sphaerospora rutili* n. sp. and *Sphaerospora* sp. ex *Rutilus rutilus*, for which no replacement was found for the host, were excluded from this analysis. Due to incomplete sphaerosporid 18S rDNA data, *Sphaerospora elopi* n. sp., *Sphaerospora* sp. ex *Gobio gobio* and *Sphaerospora* sp. ex *Scardinius erythrophthalmus* were additionally withdrawn from the analysis.
