# Supplementary material for: Biodiversity and host-parasite cophylogeny of Sphaerospora (sensu stricto) (Cnidaria: Myxozoa)
Source: Parasit Vectors. 2018 Jun 15;11:347. doi: 10.1186/s13071-018-2863-z (PMC6002976; doi:10.1186/s13071-018-2863-z)
Supplement: Supplementary file 10 — Table S9. ParaFit analysis (performed in APE package v3.4 in R v3.2.4) result for sphaerosporids and their vertebrate hosts. Statistically significant results (< 0.050) are indicated in bold. Test statistics were calculated (i) between the of sums of squares of values in the main diagonal of the combined host-parasite matrix and a matrix with unspecific interaction to estimate the ligand-receptor relation which is more restricted and preferred value (F1.stat), and (ii) the difference calculated by the trace of non-permutated matrix (F2.stat). (DOCX 34 kb) [file 13071_2018_2863_MOESM10_ESM.docx]

**Additional file 10: Table S9:** ParaFit analysis (performed in APE package v3.4 in R v3.2.4) result for sphaerosporids and their vertebrate hosts. Statistically significant results (<0.050) are indicated in bold. Test statistics were calculated i) between the sums of squares of values in the main diagonal of the combined host-parasite matrix and a matrix with unspecific interaction to estimate the ligand-receptor relation which is more restricted and preferred value (F1.stat) and, ii) the difference calculated by the trace of non-permutated matrix (F2.stat). Permutation tests were performed by arbitrary combination of values in rows of the relation matrix.

| **Host** | **Parasite** | **F1.stat** | **p.F1** | **F2.stat** | **p.F2** |
| --- | --- | --- | --- | --- | --- |
| *Sphaerospora angulata* | *Carassius auratus* | 0.01323873 | 0.073 | 0.02276865 | **0.034** |
| *Sphaerospora dykovae* | *Cyprinus carpio* | 0.005612479 | 0.059 | 0.00965263 | **0.031** |
| *Sphaerospora elegans* | *Gasterosteus aculeatus* | 0.012689108 | 0.096 | 0.021823381 | 0.05 |
| *Sphaerospora epinepheli* | *Epinephelus malabaricus* | 0.006475256 | 0.06 | 0.011136479 | **0.018** |
| *Sphaerospora formosa* | *Merlangius merlangus* | 0.007112233 | 0.084 | 0.012231984 | 0.078 |
| *Sphaerospora fugu* | *Takifugu rubripes* | 0.005214923 | **0.045** | 0.008968893 | **0.021** |
| *Sphaerospora molnari* | *Cyprinus carpio* | 0.004873117 | 0.093 | 0.008381037 | **0.047** |
| *Sphaerospora motemarini* | *Lutjanus peru* | 0.003728672 | 0.175 | 0.006412763 | 0.092 |
| *Sphaerospora ohlmacheri* | *Rana catesbeiana* | 0.009759095 | **0.011** | 0.016784195 | **0.007** |
| *Sphaerospora ranae* | *Rana catesbeiana* | 0.009581881 | **0.019** | 0.016479413 | **0.014** |
| *Sphaerospora sparidarum* | *Sparus aurata* | 0.002686399 | 0.315 | 0.004620208 | 0.169 |
| *Sphaerospora sparis* | *Sparus aurata* | 0.014089555 | 0.065 | 0.024231943 | **0.039** |
| *Sphaerospora truttae* | *Salmo trutta* | 0.020564614 | **0.002** | 0.035368082 | **0.001** |
| *Sphaerospora* sp. ex *Liza ramado* | *Liza affinis* | 0.018968966 | **0.002** | 0.032623805 | **0.001** |
| *Sphaerospora* sp. ex *Chelon labrosus* | *Chelon labrosus* | 0.00883949 | **0.012** | 0.01520261 | **0.004** |
| *Sphaerospora* sp. ex *Pomoxis nigromaculatus* | *Pomoxis nigromaculatus* | 0.019890858 | **0.001** | 0.034209322 | **0.001** |
| *Sphaerospora* sp. ex *Ptychadena anchietae* | *Ptychadena mascareniensis* | 0.019834062 | **0.002** | 0.034111642 | **0.001** |
| *Sphaerospora* sp. ex *Abramis brama* | *Abramis brama* | 0.019647542 | **0.002** | 0.033790855 | **0.001** |
| *Sphaerospora abrami* n. sp. | *Abramis brama* | 0.019505178 | **0.001** | 0.033546011 | **0.001** |
| *Sphaerospora bliccae* n. sp. | *Blicca bjoerkna* | 0.019546808 | **0.001** | 0.033617607 | **0.001** |
| *Sphaerospora* sp. ex *Ctenopharyngodo idella* | *Ctenopharyngodon idella* | 0.019654222 | **0.001** | 0.033802343 | **0.001** |
| *Sphaerospora diminuta* | *Lepomis gibbosus* | 0.010670834 | **0.006** | 0.01835225 | **0.003** |
| *Sphaerospora diversa n. sp.* (*Leuciscus idus*) | *Leuciscus idus* | 0.009474214 | **0.005** | 0.016294242 | **0.003** |
| *Sphaerospora diversa* n. sp. (*Leuciscus leuciscus*) | *Leuciscus idus* | 0.009329902 | **0.009** | 0.016046047 | **0.004** |
| *Sphaerospora* sp. ex *Lota lota* | *Lota lota* | 0.008262823 | **0.014** | 0.014210829 | **0.007** |
| *Sphaerospora* sp. ex *Sander lucioperca* | *Sander lucioperca* | 0.004290501 | 0.127 | 0.007379024 | 0.075 |
| *Sphaerospora dentata* n. sp. | *Scardinius erythrophthalmus* | 0.011759546 | 0.123 | 0.020224674 | 0.066 |
| *Sphaerospora gutta* n. sp. | *Scardinius erythrophthalmus* | 0.002826997 | 0.172 | 0.004862015 | 0.086 |
| *Sphaerospora diversa* n. sp. (*Squalius cephalus*) | *Squalius cephalus* | 0.018245638 | **0.003** | 0.031379788 | **0.001** |
| *Sphaerospora squalii* n. sp. | *Squalius cephalus* | 0.021239812 | **0.001** | 0.036529324 | **0.001** |
| *Sphaerospora* sp. ex *Silurus glanis* | *Silurus glanis* | 0.02155611 | **0.001** | 0.037073309 | **0.001** |
